# Expression of A New Endogenous Retrovirus-Associated Transcript in Hodgkin Lymphoma Cells

**DOI:** 10.3390/ijms20215320

**Published:** 2019-10-25

**Authors:** Jana Schneider, Ines Volkmer, Kristina Engel, Alexander Emmer, Martin S. Staege

**Affiliations:** 1 Department of Surgical and Conservative Pediatrics and Adolescent Medicine, Martin Luther University Halle-Wittenberg, 06097 Halle, Germany; jana.schneider79@outlook.com (J.S.); ines.volkmer@uk-halle.de (I.V.); kristina.engel@uk-halle.de (K.E.); 2 Department of Neurology, Martin Luther University Halle-Wittenberg, 06097 Halle, Germany

**Keywords:** Hodgkin lymphoma, gene expression, colony stimulating factor 1 (CSF1), endogenous retrovirus (ERV), LTR8

## Abstract

During characterization of a cDNA library from the Hodgkin lymphoma (HL) cell line L-1236, we discovered a new transcript derived from chromosome 1 at the long intergenic non-protein coding RNA 1768 (LINC01768)/colony stimulating factor 1 (CSF1) region. The first exon of this transcript from Hodgkin lymphoma cells (THOLE) starts in the predicted exon 4 of LINC01768 and is part of an endogenous retrovirus (ERV) from the HUERS-P1/LTR8 family. High expression of THOLE was only detectable in HL cell line L-1236. The expression of THOLE in L-1236 cell is another example for ERV/LTR-associated gene expression in HL cells. At the genome level, the HUERS-P1/LTR8 region including THOLE is only present in Hominoidea. The influence of ERV/LTRs on gene expression might explain the characteristic phenotype of human HL.

## 1. Introduction

Animal models are potentially powerful tools for the study of human neoplasia. In principle, two types of animal cancer models are in use. Type one includes naturally occurring cancers in animals. In this case, the tumor cells have usually a similar phenotype as in the corresponding human disease. The second group of animal models includes all types of xenografts. Xenografts have the disadvantage that the transplanted human cells grow in an organism with a background of species-specific factors that might not optimally represent the situation in the human body. Receptor–ligand interactions are often species-specific. For instance, murine interleukin 2 (IL2) has a higher affinity than human IL2 for the murine IL2 receptor and human IL2 has an extremely higher affinity than murine IL2 for the human IL2 receptor [1,2]. For IL4 this species-specificity is even higher and nearly no binding of human IL4 to the murine receptor can be detected, and vice versa [2]. “Humanized” animals cannot eradicate this problem completely because it is obviously impossible to “humanize” all potentially (and often unknown) relevant molecules in an animal.

Unfortunately, several cancer entities are known only in man and for which no natural animal models are available. One example for such a cancer entity is Hodgkin lymphoma (HL). This lymphoma has been described rarely in animals [3,4]. For instance, lymphomas are the most common malignant neoplasms in horses, but among a series of 203 cases, not a single HL was diagnosed [5]. No single genetic mutation that might be useful for the generation of animal models by genetic engineering technologies has been identified as tumor initiating event in HL. Epstein-Barr virus (EBV) has been identified as potential factor for HL pathogenesis [6,7]. Infection of “humanized” mice with EBV can lead to Hodgkin-like lymphomas [8]. In this case, the malignant cells are of human origin. Therefore, this model can be considered as a xenotransplantation model with in vivo transformation. In an HLA-class II transgenic mouse model, malignant Hodgkin-like cells have been described [9]. Naturally occurring Hodgkin-like lymphomas have been observed in the SJL/J mouse strain [10]. Interestingly, the T cell receptor repertoire of lymphoma-infiltrating T cells in these mice shows a restricted usage of V beta gene segments, suggesting that a superantigen is involved in this reaction [11].

The question remains as to why some cancer entities are present more or less exclusively in humans and not in other animals. In the case of virus-associated tumors like EBV-positive HL, host-tropism of the involved virus might be a limiting factor. However, EBV is only present in one part of human Hodgkin lymphomas [7]. Moreover, EBV (or a closely related virus) has been shown recently to be present in dogs without any association to lymphoma [12]. 

Hodgkin lymphoma is a neoplasia with a proposed B cell origin. However, the phenotype of HL cells is unique and has little similarity with normal B cells [13]. This can partially be explained by the lack of functional B cell receptors in these cells. Such cells are normally eliminated by apoptosis. Anti-apoptotic signals that help HL cells to survive in vivo are partially delivered by normal cells (“bystander cells”) that constitute the vast majority of all cells in Hodgkin lymphoma. HL is an example for the close interaction between tumor cells and the tumor stroma [14].

Endogenous retrovirus (ERV)-like elements (ERVLE) have been shown to be activated in HL cells [15,16,17,18]. For instance, expression of the colony stimulating factor 1 (CSF1) receptor (CSF1R) in HL cells is driven by a long terminal repeat (LTR) from an ERVLE [15]. CSF1, also known as macrophage colony stimulating factor (MCSF), is a cytokine that acts as growth factor and differentiation factor for myeloid cells and at least for some tumor cells [15,19].

ERVLE are a major component of the (non-coding part of the) human genome. In most cells, the majority of ERVLE is transcriptionally silent. Activation of ERVLE has been implicated in auto-immunity and cancer [20]. ERVLE are species-specific and show inter-individual variability [21]. The impact of ERVLE-associated polymorphisms on disease predisposition or therapy response has not been clarified. Moreover, the species-specificity of ERVLE might explain the observation of species-specificity of cancers like HL. 

Compared to many other oncological diseases, prognosis for HL patients is excellent. However, some patients suffer from therapy-resistant disease. In order to identify potential new therapeutic targets for HL, we characterized the gene expression profile from HL cell lines. During these investigations we established cDNA libraries from HL cell lines. The initial characterization of these libraries led to the identification of an ERVLE-associated transcribed locus in HL cells [22]. In the present study, we describe another ERVLE-associated transcript that was identified by the same approach.

## 2. Results and Discussion

**Figure 1:**
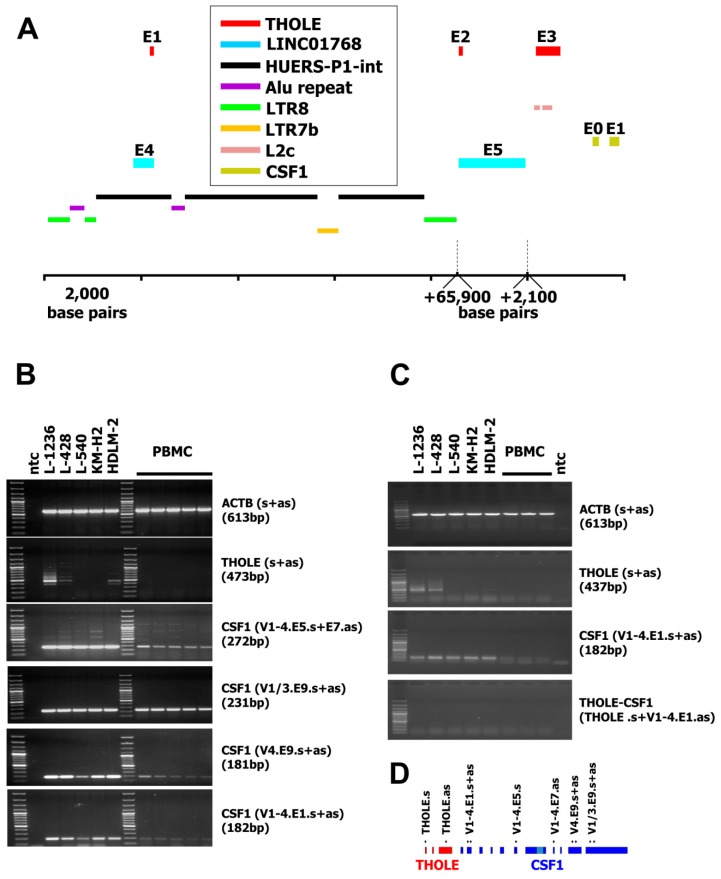
Genomic organization of the LINC01768/THOLE/CSF1 locus and expression of transcript from Hodgkin lymphoma cells (THOLE) in Hodgkin lymphoma cells. (**A**) The figure shows the location of exons (**E**) and repetitive elements in the predicted LINC01768, THOLE, and CSF1 region (genome version: GRCh38.p12; NC_000001.11; nucleotide position 109,831,234–109,931,057). Only the first two exons from CSF1, only the last two exons from LINC01768 and only repetitive elements discussed in the manuscript are shown. For size limitations a gap has been introduced between the 3′ LTR8 and exon 2 of THOLE and a second gap between exon 5 of LINC01768 and exon 3 of THOLE. The positions and sizes of these gaps are indicated by dashed lines (**B**) Presented are representative results from RT-PCR with the indicated primer combinations (s := sense primer; as := antisense primer). cDNA from five PBMC preparations from unrelated donors as well as from the indicated Hodgkin lymphoma (HL) cell lines was used as template for RT-PCR. Actin beta (ACTB) served as housekeeping control; ntc := no template control. The product size for the primer combination “CSF1 (V1-4.E5.s+E7.as)” is the size of the strong band and corresponds to transcript variant 3 of CSF1 (accession number NM_172211.3). Weaker bands correspond to transcript variants 1/4 (1166 bp) and 2 (818 bp). See also Supplementary Material. (**C**) Presented are representative results from RT-PCR with the indicated primer combinations. cDNA from three PBMC preparations as well as from the indicated HL cell lines was used as template for RT-PCR. Actin beta (ACTB) served as housekeeping control; ntc := no template control. No products were detected for the THOLE-CSF1 combination. The expected size of a spliced product including exon 1–3 from THOLE and exon one from CSF1 is 921 bp. (**D**) Schematic presentation of the primer binding positions in the THOLE/CSF1 region.

A new transcript was isolated during characterization of a cDNA library [22] from L-1236 Hodgkin lymphoma cells [22]. Sequencing of one of the vectors from the library revealed an unknown transcript from chromosome 1 between the loci for the predicted long intergenic non-protein coding RNA 1768 (LINC01768) and CSF1. The 5′ part of this transcript starts in exon 4 of the predicted LINC01768. Exon 2 is identical to the 5′ part of exon 5 from LINC01768. The third exon is located upstream of CSF1 (Figure 1A). For simplicity, we named this sequence Transcript from HOdgkin Lymphoma cElls (THOLE). HL is known for the high activity of ERVLE, and the receptor for CSF1 has been shown to be expressed in HL under control of an ERVLE [15]. Therefore, we asked whether THOLE might also be related to the activity of ERVLE. RepeatMasker analysis of the 5′ upstream region of THOLE indicated that this region indeed contains long terminal repeat sequences (LTR8 according to the nomenclature used by RepeatMasker and other data bases; Figure 1A and Supplementary Material 1–3). According to RepeatMasker analyses, this LTR8 has a length of 691 base pairs and is complete but interrupted after 453 base pairs by an Alu repeat in anti-sense orientation (312 base pairs). Similar Alu insertions are present in other members of the LTR8 family [23], and this is a typical example for a nested repetitive element [24]. LTRs are the typical promoters from ERV and LTR8 is the LTR of the so-called human endogenous retrovirus sequence P1 (HUERS-P1) [25]. Analysis of the complete genomic region between the identified LTR8 and the CSF1 locus indicated that this region contains a large HUERS-P1 copy with an internal region (HUERS-P1-int) flanked by two LTR8. The first exon of THOLE is included in the HUERS-P1-int region. HUERS-P1-int is interrupted by a second Alu repeat and an unrelated LTR7b sequence (Figure 1A and Supplementary Material 1–3). The third exon of THOLE overlaps in antisense orientation with two repetitive elements of the L2 family (Figure 1A and Supplementary Material 1–3). RT-PCR indicated high expression of THOLE in cell line L-1236 and weaker expression in HL cell lines L-428 and HDLM-2 but not in peripheral blood mononuclear cells (PBMC; Figure 1B). In contrast, CSF1 was highly expressed in all HL cell lines (Figure 1B). Whole exome sequencing data from the Sequence Read Archive (SRA) database (https://www.ncbi.nlm.nih.gov/sra/) indicate highly similar mapping of reads from different HL cell lines to the THOLE/CSF1 locus, suggesting that no larger rearrangements are present. In addition, we found no differences between LTR8 sequences from THOLE-positive HL cells and THOLE-negative HL cells (Supplementary Figure S1).

**Figure 2:**
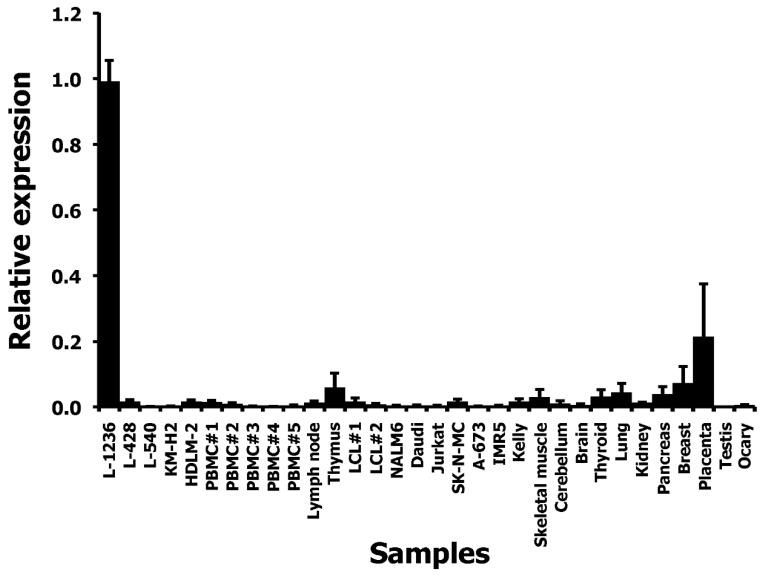
Quantification of THOLE transcripts in HL cell lines and other samples. cDNA from HL cell lines, PBMC, and a panel of normal tissues and tumor cell lines was used as template for quantitative RT-PCR. Expression in L-1236 cells was set as one. Presented are means and standard deviations from three experiments.

We asked whether THOLE-CSF1 fusion transcripts might be a source for CSF1 protein encoding RNA. Attempts to detect such fusion transcripts by RT-PCR using forward primers for specificity for THOLE and reverse primers with specificity for exon 1 of CSF1 variants 1–4 failed (Figure 1C). Whether the identified THOLE transcript is complete or whether other CSF1 exons might be included in alternative THOLE transcripts require further investigations. By qRT-PCR, high levels of THOLE were only detectable in L-1236 cells and not in other samples (Figure 2). Interestingly, other LTR8 sequences have been shown to be activated by Epstein-Barr virus (EBV) [26]. All HL cell lines analyzed in our study are EBV negative. However, HL cells express several genes usually activated by EBV or other herpes viruses, e.g., EBV-induced 3 (EBI3) [27] or interleukin 26 [28]. We found no expression of THOLE in EBV immortalized lymphoblastoid cell lines (LCL; Figure 2). We further tested a possible activation of THOLE by EBV in a conditionally EBV-immortalized cell line (EREB2-5 cells). We found no expression of THOLE in these cells independent of the EBV status (Supplementary Figure S2). 

The phenotype of cells essentially depends on the spectrum of genes that are expressed in these cells. In addition to conventional genes, genomes of all animals contain several families of repetitive elements (RE). These elements can regulate expression of adjacent conventional genes. The position of RE in the genome varies from species to species and, therefore, tumor specific reactivation of repetitive elements with subsequent expression of adjacent genes can be species-specific. It is not necessary that the genes that are expressed under control of such RE in tumor cells are involved in tumor development. It is only necessary that these factors fine-tune the phenotype of the tumor cell. If such RE are absent in one species, a tumor entity with the corresponding phenotype will be absent in this species. The LTR8/HUERS-P1 element was discovered in 1987 and it was found only in DNA from higher primates [25]. Conservation analysis of the THOLE region (Supplementary Figure S3) and CSF1 region (Supplementary Figure S4) region indicates that the HUERS-P1 region is conserved only in Hominoidea. In other mammals including rodents this conservation was not present. 

Expression of ERVLE is regulated in part by histone methylation. Inhibition of methyltransferases is able to activate ERV expression in cancer cells with subsequent induction of anti-viral pathways [29]. This so-called “viral mimicry” can be considered as a natural defense mechanism against transformed or virus-infected cells [30]. A binary switch model has been proposed for histone regulation [31]. In this model, histone phosphorylation antagonizes histone methylation and activates transcription. Interestingly, such histone phosphorylation can be mediated by the inhibitor of nuclear factor kappa B kinase complex (IKK) [32]. IKK is a component of the nuclear factor kappa B (NFKB) pathway. This pathway is highly active in HL cells and is critically involved in the malignant phenotype of these cells [33]. Recently, it was demonstrated that NFKB signaling leads to activation of the LTR of the so-called transposable human element 1 (THE1) family of repetitive elements [34]. Whether this pathway can also induce activity of other LTR families including LTR8 requires further investigation. Some exogenous viruses have developed mechanisms for inhibition of IKK-dependent histone phosphorylation [35]. It will be interesting to analyze possible interferences between ERVLE and such exogenous viruses in HL.

**Figure 3:**
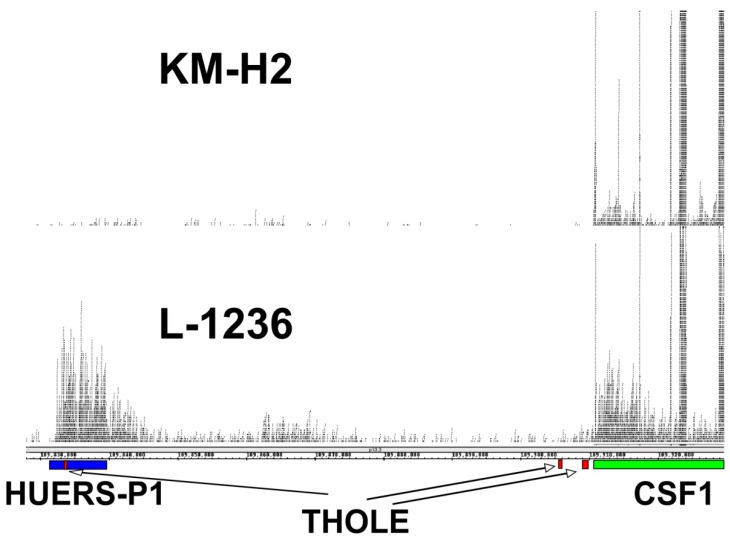
Comparison of THOLE and CSF1 transcription in HL cell lines L-1236 and KM-H2. Publicly available RNA-seq reads from HL cell lines L-1236 (SRR7890158) and KM-H2 (SRR8615908) were mapped to the human reference genome (hg38) by using Bowtie2. Mapped reads in the genomic region between the THOLE-associated HUERS-P1/LTR8 and the CSF1 locus on chromosome 1 were visualized in Integrated Genome Browser. The positions of HUERS-P1 (blue), THOLE exons (red), and the CSF1 gene (green) are indicated.

The expression of THOLE in L-1236 cells is extremely high compared to all other investigated samples. RNA-seq data from L-1236 cells and other HL cell lines (e.g., from the Cancer Cell Line Encyclopedia [36]) also demonstrate high expression of the HUERS-P1 region in L-1236 cells (Figure 3). The phenotype of the few established HL cell lines is not uniform. Among the investigated HL cell lines, L-1236 cells showed also highest expression of ERV-K transcripts [37]. ERV-K transcripts are detectable in high amounts in embryonic carcinoma cells and the embryonic transcription profile seems to correlate with the increased ERV-K expression in these cells [37]. We found moderate expression of THOLE in embryonic carcinoma cells. However, expression in these cells was lower than in L-1236 cells, suggesting that THOLE expression is regulated by other factors in HL cells (Supplementary Figure S5). Cell line L-1236 is characterized by high resistance against cytotoxic drugs [38]. The presence and potential prognostic impact of THOLE expression in clinical samples from patients with HL requires further investigation.

HL is not the only human-specific cancer. Ewing sarcoma (ES) is another interesting example. Natural animal models for ES/primitive peripheral neuroectodermal tumors (pPNET) are not available [39]. The precise nature of tumors with phenotypes similar to ES that have been anecdotally reported in dog [40,41,42], cow [43], horse [44], camel [45], and monkey [46] remains unclear. Expression of typical ES-associated markers like CD99 was not detected in these tumors [40,43]. In contrast to Hodgkin lymphoma, ES is characterized by specific oncogenic gene fusions. Attempts to model ES in mice by transgenic expression of these oncogenes failed [47]. Interestingly, transgenic expression of these oncogenes in mice usually results not in development of ES but myeloid leukemia [48]. It might be very interesting to study the possible involvement of species-specific ERVLE in this phenomenon.

## 3. Materials and Methods

### 3.1. Cells and Cell Lines

Peripheral blood mononuclear cells (PBMC) were isolated as described [49] by density gradient centrifugation from buffy coat of healthy donors with written informed consent and approval by the ethical committee of the Medical Faculty of the Martin Luther University Halle-Wittenberg, Halle, Germany. Epstein-Barr virus (EBV)-immortalized cell lines (LCL) were established as described [50] by infection of PBMC with the B95.8 EBV strain. The neuroblastoma cell line IMR5 [51] was a kind gift from F. Berthold, Cologne, Germany. Ewing sarcoma cell line A-673 [52] was obtained from the American Type Culture Collection (Manassas, VA, USA). Ewing sarcoma cell line SK-N-MC [53], neuroblastoma cell line Kelly [54], Burkitt lymphoma cell line Daudi [55], acute lymphoblastic leukemia cell lines NALM6 [56], and Jurkat [57], as well as HL cell lines L-1236, L-428, L-540, KM-H2, and HDLM-2 [58,59,60,61,62], were obtained from the Deutsche Sammlung von Mikroorganismen und Zellkulturen (Brunswick, Germany). EREB2-5 cells [63] were kindly given to us by G. Laux, Munich, Germany. NTERA-2/D1 cells [64] were a kind gift from T. Greither, Halle, Germany. Cells were cultured in RPMI-1640 medium (Invitrogen, Karlsruhe, Germany), supplemented with 10 % fetal calf serum, 100 U/mL penicillin, and 100 µg/mL streptomycin. For culture of EREB2-5 cells, medium was supplemented with 2 µM estrogen (Sigma, Heidelberg, Germany). 

### 3.2. DNA Isolation, RNA, RNA Isolation and Polymerase Chain Reaction (PCR)

DNA from Hodgkin lymphoma cell lines was isolated using the GeneJET Genomic DNA purification kit (Thermo Fisher Scientific, Waltham, MA, USA). RNA from normal tissues was purchased from Agilent (Santa Clara, CA, USA). RNA from cell lines and PBMC was isolated using the High Pure RNA Isolation Kit (Roche, Mannheim, Germany). Two µg of RNA were subjected to cDNA synthesis using 1 µL of 100 µM Oligo-dT primer (Thermo Fisher Scientific, Waltham, MA, USA). Thereafter, PCR was performed with 5 µL Green Go Taq Buffer (Promega, Mannheim, Germany), 16.8 µL water, 0.5 µL of 10 mM dNTPs (Thermo Fisher Scientific, Waltham, MA, USA), 0.25 µL of each primer combination, 2 µL cDNA, and 0.2 µL Go Taq polymerase (Promega, Mannheim, Germany). For PCR, the following sense (s) and anti-sense (as) primers have been used: avian myelocytomatosis viral oncogene homolog (MYC): MYC.s: 5′- GGC TCC TGG CAA AAG GTC A-3′, MYC.as: 5′-CTG CGT AGT TGT GCT GAT GT-3′; beta actin (ACTB): ACTB.s: 5’-GGC ATC GTG ATG GAC TCC G-3’, ACTB.as: 5’-GCT GGA AGG TGG ACA GCG A-3’; colony stimulating factor 1 (CSF1): CSF1.V1-4.E1.s: 5′-GCC TCT GGA GTG TGT GTG TC-3′, CSF1.V1-4.E1.as: 5′-GAG AGG ACC CAG GCA AAC TT-3′, CSF1.V1-4.E5.s: 5′-TCC AGT TGC TGG AGA AGG TC-3′, CSF1V1-4.E7.as: 5′-CGC TCT CTG AGG CTC TTG AT-3′, CSF1.V1/3.E9.s: 5′-CGC TGA GGA GTG AAA GAA CC-3′, CSF1.V1/3.E9.as: 5′-TAG GTG AGC CGA GGG TGT AG-3′, CSF1.V4.E9.s: 5′-CTT TGC CCA TGT TGT TGA TG-3′, CSF1.V4.E9.as: 5′-AAA GAG CAG GAG GAG CAT GA-3′; transcript from Hodgkin lymphoma cells (THOLE): THOLE.s: 5′-AGC CAC TCC ATT CTT CTG GA-3′, THOLE.as: 5′-TTG CTC AGA CTG GTC CCT CT-3′; homeobox transcription factor nanog (NANOG): NANOG.s: 5′-AAG GTC CCG GTC AAG AAA CAG-3′, NANOG.as: 5′-CTT CTG CGT CAC ACC ATT GC-3′; long terminal repeat 8 from THOLE locus (LTR8.THOLE): LTR8.THOLE.s: 5′-TTG TAG GCT GGG TGT ACG CT-3′, LTR8.THOLE.as: 5′-GTG CTG CAG ACG GGA GTT TTA-3′. PCR products were subjected to agarose gel (1.5%) electrophoresis in the presence of ethidium bromide. Quantitative RT-PCR (qRT-PCR) was performed using Go Taq qPCR master mix (Promega, Mannheim, Germany) essentially as described [65]. Relative gene expression was calculated with the 2^-∆∆Ct^ method [66]. Sequencing of PCR products was performed by using the BigDye Terminator v1.1 Cycle Sequencing Kit (Thermo Fisher Scientific, Waltham, MA, USA).

### 3.3. Isolation of A THOLE cDNA–Containing Vector

Total RNA was isolated from cell line L-1236 by using Trizol reagent (Invitrogen, Karlsruhe, Germany) and mRNA was enriched using the µMACS mRNA isolation kit (Miltenyi, Bergisch-Gladbach, Germany). A cDNA library was generated by using the pCMV-Script XR cDNA library construction kit (Agilent, Santa Clara, CA, USA) as described [22]. After plating on agarose plates, random clones were analysed by restriction digest with NotI and by sequencing using the pCMV-Script specific primers 5′-AAT TAA CCC TCA CTA AAG GG-3′ and 5’-TAA TAC GAC TCA CTA TAG GG-3’.

### 3.4. Bioinformatics Analysis

Repeats in genomic DNA were identified by using RepeatMasker (http://www.repeatmasker.org/cgi-bin/WEBRepeatMasker, last accessed date: 24. October 2019). Search for homologous sequences was performed by using BLAST [67] version 2.9.0+. RNA-seq data were down-loaded from the National Center for Biotechnology Information Sequence Read Archive (SRA). RNA-seq data were analyzed by using the Galaxy online platform [68] and visualized by using the Integrated Genome Browser [69] version 9.0.2. Species conservation of the THOLE genomic region was visualized using the UCSC genome browser (http://genome.ucsc.edu, last accessed date: 24. October 2019) [70].

## 4. Conclusions

Expression of THOLE in HL cell is another example for ERV/LTR-associated gene expression in this cell type. The impact of THOLE and other ERV/LTR-associated transcripts on the malignant phenotype of HL cells requires further investigations.

## Supplementary Materials

Supplementary materials can be found at https://www.mdpi.com/1422-0067/20/21/5320/s1.
