# Supplementary material for: Expression of A New Endogenous Retrovirus-Associated Transcript in Hodgkin Lymphoma Cells
Source: Int J Mol Sci. 2019 Oct 25;20(21):5320. doi: 10.3390/ijms20215320 (PMC6862598; doi:10.3390/ijms20215320)
Supplement: Supplementary file 1 [file ijms-20-05320-s001.pdf]

Supplementary Material for:

Schneider et al. 2019, Expression of a new endogenous retrovirus-associated transcript in Hodgkin lymphoma cells

Content:

**1. Genomic reference sequence of human chromosome 1 including THOLE and CSF1**	**Page 2**
**2. RepeatMasker results for the sequence of human chromosome 1 including THOLE and CSF1**	**Page 7**
**3. RepeatMasker alignments from the THOLE locus as discussed in the manuscript**	**Page 11**
**4. Supplementary Figure 1. Comparison of the THOLE associated LTR8 from Hodgkin lymphoma cell lines and the reference genome sequence**	**Page 23**
**5. Supplementary Figure 2. Absence of THOLE expression in EBV immortalized cells**	**Page 24**
**6. Supplementary Figure 3. Conservation of the THOLE associated ERV in Hominoidea**	**Page 25**
**7. Supplementary Figure 4. Conservation of the CSF1 locus in mammals**	**Page 26**
**8. Supplementary Figure 5. Moderate expression of THOLE in embryonic carcinoma cells NTERA-2**	**Page 27**
**9. Supplementary Figure 6. Schematic representation of the THOLE/CSF1 region with primer locations**	**Page 28**

1. Genomic reference sequence of human chromosome 1 including THOLE and CSF1 (NC_000001 REGION: 109831234..109931057)

Legend: LTR8, Alu repeat, HUERS-P1 internal region, THOLE exon, LTR7B, ***L2 repeat,*** **LINC01768 exon 4-5,** CSF1 exon

Notes:

1. Only repeats discussed in the manuscript are highlighted.
2. The first two shown CSF1 exons are used alternatively. The first exon is only used by the predicted variant XM_017000369. The two last exons are also used alternatively. The penultimate CSF1 exon is used only by transcript variant 4. Transcript variants 1 and 2 use the last exon. Transcript variants 2 and 3 use shortened versions of the 7th exon.

CAATCACATATTCATGAAATAATGTCAGGTTTTAAAAACTTTTATCAAATCAACAGAGCATTTAAATGCAATTGA
AACCGCCTTTGCAAAATTATGACTGAGACAGTGAAAGAGATCTAACCTTACTGACTCCATCTTGCTTCTAGCCTC
AAAGCTGCCCTTGTAGGCTGGGTGTACGCTGAACTAATTTGGGGGAAACTTAGTTTATAGTTTAAATAAAGATG
GTAACAGCCCTTTCCCAAAGCAGGCCTCCTTCTTGCCTGGGGACTAGATTGCCTTTGTAGGACTAACATTACCTG
CAAGATTAGAAATTATGGTTTAGGAGTCATGCAGCTGGAGACTTCAAGATTCCGATCCTCCCTAAACTGCTCCTA
AGATCATTGCTTGAGATATTTTGTAGACCCTGCGCTTGATGAATCAGCTGGCACCACCCAGATCAGTAAACTGGT
TCATCTGATCTTGTAGCCCCCACCCAGGAACTGACTCAGTGCAAGAAGACAGGTCCGACTCCCTATTATTTCTTT
CTTTCTTTCTTTTTTTTTTTTTTGAGACAGGGTCTCACACTGTTGGCCAGGCTGGAGTGCAGTGGTGCGATCTTG
GCTCACTGCAACCTCCACCTCCATATTCAAGCGATTCTCCTGCCTCAGCCTCCCGAGTAGCTGGGATTACAGGTG
GCTGCCACCACGCCCGGCTAAGTTTTATTATTTTTAATAAAGATGGGGTTTCACTATGTTGGCCAGGCTGGTCTT
GAACTCCTGACCTCGTGATCCACCTGCCTCAGCCTCCCAAAGTGCTGGGATTATAGGTGTGGGCCACTGCACCTG
GCCTGTAATTTCATCTCTGACCAATCAGCACTCCTGGCTCACTGGCTTCCCCTCACCCACCAAGTTATCCTTAAA
AACTCTGCTCCCTGAATGCTCAGGGAGACTGATTTGAGTAATAATAAAACTCCCGTCTGCAGCACAGCAGCTCTG
CATGAATTACTCTTTCACTGTTGCAATTCCCCTGTCTCAATGAATCAGCTCTGTCTAGGCAGCGGCAAGGTGAA
CCCCTTGGGCGGTTACAAATTTGGGGGCTCGTCTGGGATTGCCCTTGTGGCTACTTGCCCATGGTTCAGTGGCCT
CCTCCCCCAGCGATGAATCCAGAAGTCAGCCCAAGCGGCAGCCTGGTTCTCTTGGACTGGGGGCTGACTCTGGTA
TTTCTCTACTGGCAGGGCACTGCCGACCCGATGTGCATGGATTTAATTGCAGTGGAGAAAGAGTCCTAGGGAGAC
ACCACTTAATTGTAGCCCTATCACAGGGCGTCTGTCTGTAGCCCCATGGTGGGATGTCTGTAGCCCCATTGTGGG
CTGTCTGGATTGGTAAGTATCCTAGGCACTGCCAATGGCTTCTTCCTTCTCCTGACTGGTTATGTAGCCCTATGG
TGGTGTGTCTGTAGCACCATTGCAGGGTGTCTGTTCAGCTCCTGTGGGGGTCTCAGTTGGCTCTTTCTAACTAGT
CACACAGCTCCGAAGGTCTAATAGGAAGAGTCCTGGTTTGGGAGACTTGTCTTCAATCAGGAAGATTTCGAGGAG
TTTTCTCAGAAGGAGACTAGGAGAATAGTTTGGGAGGGATACTCTTGGAGTTCTTGGTTAGGGATCTGATTTAGA
AGGCCTTCTGTCTTTGCATGTGTTCGTATATTTGGAAGGAATCTCAGAGAGGTTGCTGATGGAAGTCCAGCAGGC
CTAACTCAGAGAACCCTCCTTATTTGTCTGGTCACATTCGATGAGCTCTAAAGAAGGCTCAACAGGCCTGTCTCA
GGGGGTGAATATCTGCTCTTCCCATTGCTCA**GATACCCCGTTGTGAATTACCATTTGGAGGTAGTCCGTCCCCAC**
CTGGAGTGGATCAAAGACAACAGAGGCCAACGGGGAAAAAGTTTGAGCTTTGCCAGGCTGATATTGGCTGCTGAA
TAAGGTGACTAATGTCTGTTTTGTTATGTGTATTTTGCTGGGATGGAAAATGTTAATTTGGTTTCCCATGCAGCC
CGTCAGGCAGCATCTTGCAAATAAAGAATCTTGTCTTACAGGGTGATTTTCTCTTGTAAAATGGCTTGAACCCCA
AAGCTATAGCACAAGCAAGCAGGGTCATCAGAAGCCACTCCATTCTTCTGGAAGCTGCAGGGAAATGGAACCCAG
AAACCAGGTATGACAGCAAAAAGGGTAAAAAATTCTTACCGGCCAAGTTTCTGATCTGTCTTGCTTTCTCTGTCT
GAATAAACGGTAAACTATTTGTCTCCTCTGCAAGGGTTTGATTAACAGAAAAAAGGATTTGTGAGACTAGTCTTA
AGCTGTAAAAATCTGGTGTGCTTTGTGCTAAGAATTTGTCTTTCTGTGTTCTGTAATGGAGAGAGGGGTATCACA
GGATAGAACATGGGTTTAGGACCCCTAAGCCTGCTTTTAAAGTCAGCTTGGCAGGCTGGTCAGTTATAAACTTTG
CTATGGGTCCCTGAAACCAATACGGTATGAAATTTCTCTGTTTTGTTTTGTGTCCTTAAGAGTTTAACCTTATGA
CCATGGGAGATACTTTCTTTTGGTTTCTACCACCCAGACGACAGGAATTTTGGGGTTCATGTCATAATCCTAATT

TTTTTTTTTTTTTTTTTTTGAGATGGAGTCTTGCTCCCATTACGAGGGCTGGAGTGCAGTGGTGCAATCTCAGCT
GAATGCAACCTCTGCCTCCCGGGGGTATTCCTTCCTCAGCCTCCCCAGCAGCTGGGATTACAATTTTTGTATTTT
TAGTAGAGACAGTGTTTCACCATGTTGGCCAGGCCGGTATCGAACTCCTGACCTCAAGTGATCCACCCGCCTCAG
CCTCCCAAAGTGCTAGGATTACAGACAGGCGTGAGCCACTGTGCCCAGCCTAAAAATTTTTCTTGAGCAATTAAA
AGCCTTGCAAGCTTGAAATTGGCTTCTCTAGGCTCCTTCTGGGAAAAGCAATAGGAACTTCTCAATGCTGTATAG
CTCGGTAGCTAAGGCTTTATCTTTTGACACTGGTGGCCTGGGTTCAATTGTTGGCTGCTGAAATGATTCATTTCT
GGTTTGTTATTTGTGTAACGTTGCCATTTATTGAGGTTTTTTCCCATCGTAGATAGCTTCTGATTTCCTCTCTTG
AATTTTCCTTTCTCTGAACTACCTTGTGGAGATTCTAAATCTTATAAAAAAAAGAAACTGCTTACCATGTCTTTG
AAGCACCTAGGAGGTTACATTTGGTAAAGTTCAGAAGCTAGAAGTATTGGCCACTTGGCATGGCTAAAGTCAGGT
AATAAGAGATTTGAAAAGATTTCTTTTTTAAAGAGCACTATGGTTAAAAGTTAGGTTAATTAAAAGTGGATAAAC
AAGCTATAGATGTATTTAAAAGGCCATTGTTTTTCTCTTCTTGGAACTTGTTTTTCTGGAAAAAGGTTTTTCCTT
CTAAGTCGACTGAACTATTTTTCTCCATTTTTTTGTCTTGCCACTCTTAATACACACATCAGAGCCCCTAAGATA
ACTTCTGGTAGCTTGGGACTCCTTGGGAAAAACAGAGGAGGTGCTGCAGACCCCGCTTTGGGGAAAAAAACCCAA
AACACTCTGTTTTCATCATGAAACCCCAGGAATTAATAGCGGATAGATCCCTCTCAAAATCAAAGGCTTGTTCTG
TTTTGCATTGTGTTGTCTGACGGTTTTGAGTTTGGGGGGCATCAGAAATTACTTTGCATTATGAGAGAGCTTTGG
TGTGTAATAACTAGGTAGGAAATATACTCTAAGGGATGGCTAATAGTAGTTAGGGAGGGGTACTTGACTCTTTGC
ACACTTGGATCAGAGAAGCATGCTCTTGGCCACCTGGAAGATAAGGAGACATCCCCACCCCCCACTGGGAGATGA
GACTCTCATGAGGGATGGGCTGATTGCAAAATGGGCTGATTGGCTTTGGGTTGCTTTGCAATGAAATGCAGGGTA
GAAGCACTGCACTGTCCTCTCCAGTAGTATTTCCCTCCTTTTGGGATCCAGGATCAGTATAAAATGGCACCCTTA
ATTTTGGGGATGTGTCTCTGCCTTCAGCTGCTTATTTGCTGCTTATTTGGCCTTAGAAATGCATGCTTTCCAGGC
CCTGTTCCTCCAAGGGCTCCACCCCGAAGCCAGTAATCCAATTAAGAAACTGGCAAATGAAAAACCTTACAAGTG
CCTAATCTTCTCTCTGTATATTTATATGTGTTGTATGTTTAGATATAAAAGAGCTCTGATTAATTGGCTTAGAAA
AATAAGCGCTTAAATCAAATATTTTGTCAGAAAAATAGAAATTTTAATGCCTTTTTTTCTCGTCATTTTAGTAAT
CTTTTGGAAATAAAGACAGTTTTAAAGATTATTGGTAAAATAAAATGTCTTGAAAATGTAGACATTTGGTCTAAA
TTAAGATCAGGTATCAGACTTGCTAAATATTTTAAGTTCAAACTGTTTCTTTGACTTTTGAAAATTGTTCGATTT
ACCTACTTTGGAGCATTAGATTATAGATAATTCCTGCGGACATGGGGAGAGCCATGTCCGCTAGCTATGCTAAAA
GGAGTCAGACCTTATCTTCATTTCTGTCTGATGTCCTAGGCTCCACCCCTAGTACATAATTAAAATCGCTTACTT
GTCAGGTTTTTCACTAAAAATAAAAGGTGCTAAGAGTTAACATTGCAACATGTAGTCGAGACCGCTGGAGAAACA
GTTTTACATACAAGGTGTGTAGGGAATGTGTTTTAGGTAAAAGATTATAAGAAGGCATGGGAATATGGCTTTTGT
TAAAGGGAATGTAATTTTGTCTAGTTCAGAGGGTTTTAAAGATTGTCTTAACCTAAAAGAGTAATGGAACAAAAC
TGAAGGTTTAAGCAAAGTGAAAAGGGTTTGAAAAGGGTTGATCTTGTAAAACTTCTGTGGGTATAAACAAGTTGG
CTAAGATTTAAAAGAAATTATTTAGCTTTTCCCCATAGGTTAAAACATTAAAATCATACTGATGTGGGGCCAGAA
TCTGGGCCCATGTGTACGAATAACAGGGTTTTCTTAGAAAATTGATCTGCTGTTTGATGGAAAATTGTAAAGGGT
TCTAAAAAGTTTATGAAAATCTTGCCTTATGGTGAAACTAATTAAAACTGGATAGAGATATAAAATTTTATCTAA
AAAACTAGCTTTAACGTTAAAAGATGCACTAATGCAAACAAGAAATTTGGTTTTCTCTTTTGAAGATGATTTTTA
TGTAATGTTAAAAGATAATGAAAGGGTTTTGTTTTCTCCTTTGGGTAAATGGCAGGGAAAAAAAAGAGGAGAGAG
ATAAGAGACAGATTGAGTTGGCCTCATGCTATCTTCATTGGGTCTTGTTTGGAAAGCTAAGTCTTCTCTATCAGA
GTGAAGGTTTTTCTTTTTTAAAAATTGTTTTGGAGTTATCAATTCGGCCAAATGAATGATTTACGGTGATCTGGG
ATCTATTTTGTGATATCCTGTGTTTTAAACCTTTGATATTTGACAAGCTTTCCAATATCAAATTATAAATTCTGT
CTCTTTCTAACCTAATATTTTAGATATTAGGTTCTTTAAAGTCCAAAAATGACATTTGGCTTATTTGGTACAAAA
ATCACACAGGAAGCATTGTCTGTCTCATGCGTCCATGTGAACAGACCACCAAACAGGCTTTGTGTGAGCAACAAG
GCTGTTTATTTCACCTGGGCGCAGGCGGTCTGAGTCTGAAAAGAGAGTCAGCGAAGGGTGGTGGGATTATCGTTA
GTTCTTATAGGTTTTGGATAGGCGGTGGAGTTAGGAGCAATGTTTTTCGGGCAGGGGGTGGATCTCACAAAGTA
AGTTCTCAAGGGTGGGGAGAATTACAAAGAACCTTCCTAAGGTGGGGGAAATTACAAAGTACGTTGATCACTTAG
GGTGGGGCAGAAACAAATCACAATGGTGGAATGTCATCAGTTAAGGCTATTTTCACTTCTTTTGTGGATCTTCAG
TTGCTTCAGGCCATCTGGATGTATACGTGCAGGTCACAGGGCATATGATGGCTTAGCTTGGGCTCAGAGGCCTGA
CATTGTCAAATATGAAATGGTGTTTGGCTTTCTTTGGTCTCTATTTGTGTAAATGTGTTATTGGTATATGTTCTG
AAGTAATGTAAAACTCCTATAATTCTAATATGACTTAGTCTATGTTATCTGTAATAATTATAATTATTATGTTAA
CAGACTGTGTGCTACAAAGGTAAATTTCCTTGTCAATTGTGTCTTTAACTGTGGCTGCCCTAAAATGTTTTTGTC
ATCCACAGATAATTGCTGTCTCGTTTTGGTCCTCTTTAAAAGATGGTTTTATAATCAGTTTTAAAATGTAACAGG
TGCTCTTAAATGCAGGATTCTGATAAATAACTCTGGAGATTGTGACATTTGAATAGAGGAAAAACTTTCAAATAG
AAGAGTGAAGGGTGTTTGGTTCACTTTGGACTGTATTTGTATAAATATATTATTAGTATGTATTCCAAAATTATG
GGAAACTTCTACAATGTTGATATAATTTAGTGTACATTGATAATAATATTGTTATGTAAAATTGTATGCCATAGA
AGTAATCAAAATTCCTAGTCAATTGTAGCTTTAATAGGGCTTTAAAATTTGTGCTATTGGAATAGAGAAAAAAAC

CAAACTTCTAGGACTGTCATGGAGAGCTAATGTGTTAAACATTGCTAAACATTTTGTTTCCAGAGTCAAGGGAAC
TTATTTCTTTAGAGGTATTTGCAACTTTTAACAAATGAATAAGATATACTCCTGAGAACAAAATTTGGAGCATAT
TTGTTCCTCTCTACCTGATTTCTCCAGAATTTGGAAATTATTTGTGAATATTCTCAATTTATGGCAGTATAGTTA
ATTGTGTAAGTGCAATAAGAAGCTATTTTCTTCTGTAACAGAACACAGTTGGAGAAACTGATTATTTTACCAAGG
TTTTGAATGGAATGGCATGCTTCCTTTAAAGAATCAAAGTTGACTTATAGAGCCAATTAAAGCCTGTTGGGGAAT
CTGACCTCATACCTTGTCTGCACAGTGTCCCCGTACAAGGTTCCTGACCTGTGGTAAGTAAGGAATTTCACTTTC
TAACAGGCCCAGGAACCCCAAGTTGTCTTGGGACCTCAAGAGGAGAGGAATTTGCCCAACTCATAGGTATTTGTC
GGTACAAACCCACAGCTGGGCTTGGCTTTTAAAAAGTCTTATCTGAGATTCTTCATGGAACAGAGTTCTATCAAA
GCCAATTAAAAAAGTCTACATGAAAAATAATTATTCTTGCTGTACTTTTTACAAATAATTAGGCCAATTACAGTA
AGACTAAAGTTTATTTTGTAAACGAATCAGTTCTATCATGATTTGTTTTTAAAAAAATGAAGACTGGAGAGAGAA
AATTTATGCTTCAAAAGAAAAACTATAGTACACTGTTGTTAGCTATTCTTGAGGTTTTTTTCTGTAGTTTAGACT
AAATTCCAAATTCTTTGTGGGCTAGAAGTCCTCAAACTAATGCTTTCAAATCTTTGTTTTTAAAATTGGGAATTG
TACTCCTCAGCCTAGGACTCATTATTTACCTTATAATAGGCTGTTTCACTTAAACACTGTAGTAAAACTATAGTT
GAGAGTACTAATGTTTTTGCCATGAAAGCCCAGCCAGGCCTGCATGAGTCACTCAGACAGTTACAAAGCAGTTCA
ACTCTTCTCACCTTGGGATGCACTCCCATTTCCACTACGTCCTCTGTTGGCAGGAAGAAGCCAGAGCGATTTACA
ACCTTTTCCCATCTTCATAGCCTACATCGTAAGACGAGGGCATTATAAAACCCAAAGGGAAGGATTGAAACTGCC
TTTGCAATATTATGACTGAGACAGTGAAAGAGATCTAAGTATACTGACTCCATCTTGTTTCTAACCTCCAAGCTG
TCCTTGTAGGCTGGGCATAGACTGAACTAACTTTGGGAAAAAACTTAGTTTATAGTTTAAACAAAGATGGTAACA
GCCCTTTCCCAAAGCAGGCCTCCTTCTTGCATGGGAACTAGATTGCCTTTGTAGGACTAACATTAGCTGCAAGAT
TAGAAATTATGGTTTAGGAGTCATGCAGCTGGAGGCTACAAGATTCTGAGCCTCCCTAAATAGCTTCCAAGATCA
GAGCTTGAGATATTTTGCAGACCCTGCACTTAATGGATCAGCTGGCACCACTAAGATCAATAAACTGGCTCATCT
GATCTTGTGACTCCCACCCAGGAACTGACTCAGTGCAAGAAGACAGGTCCGACTCCCTATGATTTCATCTCTGAC
CAATCAGCACTCCTGGCTCACTGGCTTAGTTATCTTTAAAAACTCTGCTCCCCAAATGCTCAGGGAGACTGATTT
GAGTAATAATAAAACTCTGGTCTCCCACATAGCTGGCTCTGCGTGAATTACTCTTTCTCTATTGCAATTCACCTG
TCTTGATGAATCGGCTCTGTCTAGGCAGCAGGCAAAGCAACTTTGGGTGGTTACACAATGAAAATATTAAGAAAA

[+65,850 base pairs]

TTCGGTTACACAGCTAATGCTCTTACTCACCACACTGCACTCGTGAGGGCTGTGAGACTCTTCCTTCCCCCAG**GG
AGAAGTGCCCAGCAGCAGCCAATGGAAAGGGGACTTGCCTGCTGGCTGGAGGACCCCGGCCTGGGGCTCACGGCA
GCCACAGAAATGAGTGAGTATTGCTGGTTGTAGTTGGAGTCACCAATGCCTTTCCTTCCTTCACATGGGGCAACC
TTAGGAAGGTGTCCTCTCCCCACTTACTCTCCTGCTCTGGGCCTCCCCCATCTCTGCCCATCCTGGCCTCCATAC
AGAGCATCCAAGCATCACCTTGGACCTCCCAGTTGGCACTGGGCAAACCAACATAGAGAACCAAGACAAAGCACA
ACCTTAAATCATGTAAGTGGAATAGAGGAGTCTGTCTCTCTGGCTGTATCTAGGACCTGATTTCTTCTAATTCCC
AATCTCTTCTCTACTCCAGGTTTCTGATTTCCTTCAAGTGACTTCTGAAGCCAAGCCACTCTGGGCCTTTGGGGC
ACTGGAACATTTCTTCATTGTCCTACTCACTGCCCCTTGATGTGTGTGTCCCTGGCAGTCTGTGAGGAGGGAGGC
CATGTAATTCAACGAAGTGTGGGCAAGCACAGGCCAGGCATGCAGCAGGCACCAAAAATGTCTATTGTACTAATA
GATGACTACAGACAGTGTAGGTTCTCACTTCCTGTTTCTAGGTAATTCTCTGCCTCGTTTACTCTGAAGTCACAC
AGGGCCAGGATAGGAAGTGGGCTTGCTGTGATGCTTGTGCACCAGCTGACACATCATCCTTTTCGTGATTCTCTT
TTTCCGATGAAGGTTGGAAACTTTCATTCTAGAAGGGTCCTCAGCTGAGGGATTGTGACTTTAGGGAGGTAGAGA
GGGCACTTGGGAAGCCAGAATTCACTGTGGATAAATGTTTGTCAGTTTCTGGGTTAGATTTTTCAGTTCTGAGGA
GTTCATGAGAGCAACCTAAGAATCAGTCCTGCTGAGGTTCAGGAGACAGTGCCGGGCCTGGCTGTGGCGGGAAGC
TCCGGGAGAGGAAAGTGGACAGGCTGCTGATACAGTCTTGCATTGCAGCAGTGCTTGCCCTTCAGGGGTGACACA
AGTCCTGAAGCCACCATGTATTTCTGTGGCTCTGGGACAAGGGGGACAGCTGACTTTCCCCCAGCCATAGGATGG
GGTGGTCTCGACAAATATCAATAGTTCCAAAGTACCAAAGATTGGCTCTTGGTAGTTTGCCAAACACAAAGGCTT
CACAGCAGCCCTAGGAAGCTGGCAGGAAGCTGCTATTCCCATTTAATTGACAAGGGCATTCAGTCCAAAATCCTT
CCCTGCTGGCTGCTTTACCACCCTTCACGCTGTCTCTCACCTTGAAAGTGGGAGAAGACATGACTGAAAGGGCAG
GCTGCAGTGCAAATGACTATAGGTGCGTGGCTGGGA**GATGGGATGCTCTGGCTCTGACGTCCTGCTCCCAAACCT

[+2,175 base pairs]

TGCCTAGCATCTGGTAGCACATATTTGTCAAAAGAACAAAGTATTCTCTAATGGCA***TCATTCAACTCAGCCATCA
TTAACTTGGTACCTATTATGTGCTAGGTACCATTGATCCAGAGGTGACTGAGCATGGGTGCCACCTGGAAGAAGT
CTCGGTCTAGGGTGGGGACAGAATGTAACCA***TCGGTACCACCTTAAACACTTCGAAAAGTACTGTGGAGAATGAA
TGGATGG***CAAATGACTTATCAGAGCCAGCATTGAGGAATGAGCCAAGTCCAATGGGAACACAGGAAAAGGACCCT***

<u>TTGCTCTGCCAGGAGAGTCACAGAAGGCTTTGCTGGTGGAGGATGAGATGCAATTTCAGCCTGAAATGATGAGGA</u>
<u>GTTTGTCTTCAGCCATGTGGAGAAAGGAGCATTCCAGGCAGAGGGACCAGTCTGAGCAAAGGC</u>ATTCGGGTGTAA
AATTATTGCCTAGGATTGGAGTGAAGGATATGTAGCGCTACTGACAAGAGACTCCTCTTCAAGAGCAGGCCACAT
ATCCTTTATTTCCCAGGGGGTGGAAACGGCAACCTCACAGAGGAATTGAATCATAATGAATTGAAGAAAGAAAA
ATCTCCTGAAGTTGGCATTATAATTAATAGGCTTAGGCTCATCCTACTAGTCCTCACTTAGACTCATTCTCATGT

[+525 base pairs]

TTCTGCTAAGATTTGGGGATTTTCAGGCCTGGAGGGAAAGTCCCTTGGGACGATCATAGAGCGCTAGCACTGAAT
CAGCCTGGAGAGCGCGGAAGGAAAGGGTCGGTCCGCAGAGGGCGCGGGGAAGGCAGGGTGGGGACGCGGTGGAGC
CCGCGCTCGTTTGCTGAAGGCTTGGAAGTGCAGCGCAGAAGACAGAGGGTGACTAGGAAGACGCGCGAGCGGGGC

[+150 base pairs]

CAGTCCTCTCTTAAAAGGCTGTGCCGAGGGCTGGCCAGTGAGGCTCGGCCCGGGGAAAGTGAAAGTTTGCCTGGG
TCCTCTCGGCGCCAGAGCCGCTCTCCGCATCCCAGGACAGCGGTGCGGCCCTCGGCCGGGGCGCCCACTCCGCAG
CAGCCAGCGAGCGAGCGAGCGAGCGAGGGCGGCCGACGCGCCCGGCCGGGACCCAGCTGCCCGTATGACCGCGCC
GGGCGCCGCCGGGCGCTGCCCTCCCACGGTAAGCGACGGCCGCGGCGCTGGGCCCGGGACGGGCTGGGGCGGGGG

[+3,075 base paires]

TCTGCGTTGAGCAGGGCATGGGGATAACTGGGGAGAGTGAGACCTGGGGAGAAATGACACCCTCTCTGTCACAGA
CATGGCTGGGCTCCCTGCTGTTGTTGGTCTGTCTCCTGGCGAGCAGGAGTATCACCGAGGAGGTGTCGGAGTACT
GTAGCCACATGATTGGGAGTGGACACCTGCAGTCTCTGCAGCGGCTGGTGAGTGTGTGGCCATGCTGTATTCTAC

[+1,200 base pairs]

TCGTTGGCCTGCTCTCTCTTACAGATTGACAGTCAGATGGAGACCTCGTGCCAAATTACATTTGAGTTTGTAGAC
CAGGAACAGTTGGTGAGTGATGGCTTTTTACAAAATCCATGCACCAGCCTGCATGCAACTCCCAGGGTGGGGTGT

[+1,500 base pairs]

CCAGGCCATAAGCTCCAGGTTCCTGTTTTTCAGAAAGATCCAGTGTGCTACCTTAAGAAGGCATTTCTCCTGGTA
CAAGACATAATGGAGGACACCATGCGCTTCAGAGATAACACCCCCAATGCCATCGCCATTGTGCAGCTGCAGGAA
CTCTCTTTGAGGCTGAAGAGCTGCTTCACCAAGGATTATGAAGAGCATGACAAGGTAGGAAGCCCTGAGGCCTGG

[+4,350 base pairs]

ATCTCCTTCCAGGCCTGCGTCCGAACTTTCTATGAGACACCTCTCCAGTTGCTGGAGAAGGTCAAGAATGTCTTT
AATGAAACAAAGAATCTCCTTGACAAGGACTGGAATATTTTCAGCAAGAACTGCAACAACAGCTTTGCTGAATGC
TCCAGCCAAGGTAAGCATGGCAGGGGCCAGCAAGTGTGTGGGGGTGGTAGCATATGGAATGGGGATTGGGAGGTG

[+1,050 base pairs]

TAGTGACTCTATCTCCTCCCCATCTTTCTCTCTCCTTCTCTCTGTGGTTCTTTCAGATGTGGTGACCAAGCCTGA
TTGCAACTGCCTGTACCCCAAAGCCATCCCTAGCAGTGACCCGGCCTCTGTCTCCCCTCATCAGCCCCTCGCCCC
CTCCATGGCCCCTGTGGCTGGCTTGACCTGGGAGGACTCTGAGGGAACTGAGGGCAGCTCCCTCTTGCCTGGTGA
GCAGCCCCTGCACACAGTGGATCCAGGCAGTGCCAAGCAGCGGCCACCCAGGAGCACCTGCCAGAGCTTTGAGCC
GCCAGAGACCCCAGTTGTCAAGGACAGCACCATCGGTGGCTCACCACAGCCTCGCCCCTCTGTCGGGGCCTTCAA
CCCCGGGATGGAGGATATTCTTGACTCTGCAATGGGCACTAATTGGGTCCCAGAAGAAGCCTCTGGAGAGGCCAG
TGAGATTCCCGTACCCCAAGGGACAGAGCTTTCCCCCTCCAGGCCAGGAGGGGGCAGCATGCAGACAGAGCCCGC
CAGACCCAGCAACTTCCTCTCAGCATCTTCTCCACTCCCTGCATCAGCAAAGGGCCAACAGCCGGCAGATGTAAC
TGGTACCGCCTTGCCCAGGGTGGGCCCCGTGAGGCCCACTGGCCAGGACTGGAATCACACCCCCCAGAAGACAGA
CCATCCATCTGCCCTGCTCAGAGACCCCCCGGAGCCAGGCTCTCCCAGGATCTCATCACTGCGCCCCCAGGGCCT
CAGCAACCCCTCCACCCTCTCTGCTCAGCCACAGCTTTCCAGAAGCCACTCCTCGGGCAGCGTGCTGCCCCTTGG
GGAGCTGGAGGGCAGGAGGAGCACCAGGGATCGGAGGAGCCCCGCAGAGCCAGAAGGAGGACCAGCAAGTGAAGG
GGCAGCCAGGCCCCTGCCCCGTTTTAACTCCGTTCCTTTGACTGACACAGGCCATGAGAGGCAGTCCGAGGGATC

CTTCAGCCCGCAGCTCCAGGAGTCTGTCTTCCACCTGCTGGTGCCCAGTGTCATCCTGGTCTTGCTGGCCGTCGG
AGGCCTCTTGTTCTACAGGTGGAGGCGGCGGGTGAGTAGATCCCCATGAGGAAGAAGAGCACGTCCCTTAGGGCA

[+525 base pairs]

TCTTTCCTGTCCTCAGAGCCATCAAGAGCCTCAGAGAGCGGATTCTCCCTTGGAGCAACCAGAGGGCAGGTGAGA

[+300 base pairs]

CTCTGCCTGCAGCCCCCTGACTCAGGATGACAGACAGGTGGAACTGCCAGTGTAGAGGGAATTCTAAGGTAAGAT

[+825 base pairs]

AACGTTCCTTATATGATATGGTTTTCAGACCCCTCACCATCCTGGACACACTCGTTTGTCAATGTCCCTCTGAAA
ATGTGACGCCCAGCCCCGGACACAGTACTCCAGATGTTGTCTGACCAGCTCAGAGAGAGTACAGTGGGACTGTTA
CCTTCCTTGATATGGACAGTATTCTTCTATTTGTGCAGATTAAGATTGCATTAGTTTTTTCTTAACAACTGCAT
CATACTGTTGTCATATGTTGAGCCTGTGGTCTATTAAAACCCCTAGTTCCATTTCCCATAAACTTCTGTCAAGCC
AGACCATCTCTACCCTGTACTTGGACAACTTAACTTTTTTAACCAAAGTGCAGTTTATGTTCACCTTTGTTAAAG
CCACCTTGTTGGTTTCTGCCCATCACCTGAACCTACTGAAGTTGTGTGAAATCCTAATTCTGTCATCTCCGTAGC
CCTCCCAGTTGTGCCTCCTGCACATTGATGAGTGCCTGCTGTTGTCTTTGCCCATGTTGTTGATGTAGCTGTGAC
CCTATTGTTCCTCACCCCTGCCCCCCGCCAACCCCAGCTGGCCCACCTCTTCCCCCTCCCACCCAAGCCCACAGC
CAGCCCATCAGGAAGCCTTCCTGGCTTCTCCACAACCTTCTGACTGCTCTTTTCAGTCATGCTCCTCCTGCTCTT
TTGTATTTGGCTAATAGTATATCAATTTGCACTTATTTGGATGTTGTTTTCATATTTTTTAAAATCTTATTTTAT

[+2,025 base pairs]

GCATTCCCACTCTGCTGACTAGCCTTTCCTTCTCTCCCACAGCTGGACGCACAGAACAGTCTCTCCGTGGGAGGA
GACATTATGGGGCGTCCACCACCACCCCTCCCTGGCCATCCTCCTGGAATGTGGTCTGCCCTCCACCAGAGCTCC
TGCCTGCCAGGACTGGACCAGAGCAGCCAGGCTGGGGCCCCTCTGTCTCAACCCGCAGACCCTTGACTGAATGAG
AGAGGCCAGAGGATGCTCCCCATGCTGCCACTATTTATTGTGAGCCCTGGAGGCTCCCATGTGCTTGAGGAAGGC
TGGTGAGCCCGGCTCAGGACCCTCTTCCCTCAGGGGCTGCACCCTCCTCTCACTCCCTTCCATGCCGGAACCCAG
GCCAGGGACCCACCGGCCTGTGGTTTGTGGGAAAGCAGGGTGGACGCTGAGGAGTGAAAGAACCCTGCACCCAGA
GGGCCTGCCTGGTGCCAAGGTATCCCAGCCTGGACAGGCATGGACCTGTCTCCAGAGAGAGGAGCCTGAAGTTCG
TGGGGCGGGACAGCGTCGGCCTGATTTCCCGTAAAGGTGTGCAGCCTGAGAGACGGGAAGAGGAGGCCTCTGGAC
CTGCTGGTCTGCACTGACAGCCTGAAGGGTCTACACCCTCGGCTCACCTAAGTGCCCTGTGCTGGTTGCCAGGCG
CAGAGGGGAGGCCAGCCCTGCCCTCAGGACCTGCCTGACCTGCCAGTGATGCCAAGAGGGGGATCAAGCACTGGC
CTCTGCCCCTCCTCCTTCCAGCACCTGCCAGAGCTTCTCCAGGAGGCCAAGCAGAGGCTCCCCTCATGAAGGAAG
CCATTGCACTGTGAACACTGTACCTGCCTGCTGAACAGCCTGCCCCCGTCCATCCATGAGCCAGCATCCGTCCGT
CCTCCACTCTCCAGCCTCTCCCCAGCCTCCTGCACTGAGCTGGCCTCACCAGTCGACTGAGGGAGCCCCTCAGCC
CTGACCTTCTCCTGACCTGGCCTTTGACTCCCCGGAGTGGAGTGGGGTGGGAGAACCTCCTGGGCCGCCAGCCAG
AGCCGGTCTTTAGGCTGTGTTGTTCGCCCAGGTTTCTGCATCTTGCACTTTGACATTCCCAAGAGGGAAGGGACT
AGTGGGAGAGAGCAAGGGAGGGGAGGGCACAGACAGAGAGGCTACAGGGCGAGCTCTGACTGAAGATGGGCCTTT
GAAATATAGGTATGCACCTGAGGTTGGGGGAGGGTCTGCACTCCCAAACCCCAGCGCAGTGTCCTTTCCCTGCTG
CCGACAGGAACCTGGGGCTGAACAGGTTATCCCTGTCAGGAGCCCTGGACTGGGCTGCATCTCAGCCCCACCTGC
ATGGTATCCAGCTCCCATCCACTTCTCACCCTTCTTTCCTCCTGACCTTGGTCAGCAGTGATGACCTCCAACTCT
CACCCACCCCCTCTACCATCACCTCTAACCAGGCAAGCCAGGGTGGGAGAGCAATCAGGAGAGCCAGGCCTCAGC
TTCCAATGCCTGGAGGGCCTCCACTTTGTGGCCAGCCTGTGGTGGTGGCTCTGAGGCCTAGGCAACGAGCGACAG
GGCTGCCAGTTGCCCCTGGGTTCCTTTGTGCTGCTGTGTGCCTCCTCTCCTGCCGCCCTTTGTCCTCCGCTAAGA
GACCCTGCCCTACCTGGCCGCTGGGCCCCGTGACTTTCCCTTCCTGCCCAGGAAAGTGAGGGTCGGCTGGCCCCA
CCTTCCCTGTCCTGATGCCGACAGCTTAGGGAAGGGCAGTGAACTTGCATATGGGGCTTAGCCTTCTAGTCACAG
CCTCTATATTTGATGCTAGAAAACACATATTTTTAAATGGAAGAAAAATAAAAAGGCATTCCCCCTTCATCCCCC
TACCTTAAACATATAATATTTTAAAGGTCAAAAAAGCAATCCAACCCACTGCAGAAGCTCTTTTTGAGCACTTGG
TGGCATCAGAGCAGGAGGAGCCCCAGAGCCACCTCTGGTGTCCCCCCAGGCTACCTGCTCAGGAACCCCTTCTGT
TCTCTGAGAAGTCAAGAGAGGACATTGGCTCACGCACTGTGAGATTTTGTTTTTATACTTGGAAGTGGTGAATTA
TTTTATATAAAGTCATTTAAATATCTATTTAAAAGATAGGAAGCTGCTTATATATTTAATAATAAAAGAAGTGCA
CAAGCTGCCATGTGAGTCGTGGGAAAGTTGCTTTTGGGGTGTGGGTGGCAAACATCAGGGTGGGTGGGCAGCA

2. RepeatMasker results for the sequence of human chromosome 1 including THOLE and CSF1 (NC_000001 REGION: 109831234..109931057)

Repeats discussed in the manuscript are highlighted yellow (LTR8/HUERS-P1) and orange (L2c).

				position in query					position in repeat			
Smith-Waterman score	% div.	% del.	% ins.	begin	end	orientation	matching repeat	repeat class/family	(left) begin	end end	begin (left)	linkage id
5025	9.6	1.6	0.9	73	518	+	LTR8	LTR/ERV1	1	453	-238	1
2211	11.7	1	0.3	519	828	C	AluSx	SINE/Alu	0	312	1	2
5025	9.6	1.6	0.9	829	1067	+	LTR8	LTR/ERV1	454	691	0	1
36461	7.4	2.1	0.6	1068	2623	+	HUERS-P1-int	LTR/ERV1	1	1624	-4639	1
1655	11.7	11.2	1.6	2624	2900	C	AluSx	SINE/Alu	-9	303	1	3
36461	7.4	2.1	0.6	2901	5645	+	HUERS-P1-int	LTR/ERV1	1625	4368	-1895	1
3466	4.2	7.4	0	5646	6077	C	LTR7B	LTR/ERV1	0	464	1	4
36461	8.7	1.4	1.2	6078	7865	+	HUERS-P1-int	LTR/ERV1	4369	6263	0	1
4851	9.6	4.1	0.1	7866	8530	+	LTR8	LTR/ERV1	1	691	0	1
380	25	4.7	5.6	8811	8937	C	MER112	DNA/hAT-Charlie	-32	229	104	5
238	23.8	17.5	0	8941	9020	+	Charlie10	DNA/hAT-Charlie	1	94	-2728	6
4629	11.7	7.3	1.2	9033	9786	+	L1MB8	LINE/L1	5379	6177	-1	7
2370	8.4	0	1	9797	10109	+	AluY	SINE/Alu	1	310	-1	8
538	21.8	3.5	5.8	10124	10264	+	MER5C1	DNA/hAT-Charlie	98	235	-28	9
1659	11.8	0	0	10266	10486	+	AluSx	SINE/Alu	78	298	-14	10
16126	1.6	0.1	0	10557	13150	+	L1PA3	LINE/L1	3560	6155	0	11
326	23	4	0	13177	13250	C	MER112	DNA/hAT-Charlie	-184	77	1	5
1024	24.9	1.5	0.8	14531	14789	+	MIR	SINE/MIR	2	262	0	12
257	30.7	5.7	0	14913	15000	C	MIRb	SINE/MIR	-69	199	107	13
460	13.5	2.7	1.3	15191	15263	+	MER84	LTR/ERV1	1	74	-433	14
2888	12.5	4.3	6.8	15264	15435	+	MER84	LTR/ERV1	2	146	-361	15
2151	11.6	0.7	2	15436	15743	C	AluSx	SINE/Alu	-8	304	1	16
2888	12.5	4.3	6.8	15744	16084	+	MER84	LTR/ERV1	147	503	-4	15
472	31.3	0.8	0	16488	16618	C	MamGypLTR3a	LTR/Gypsy	-542	439	308	17
1993	16.9	0.3	0	16757	17063	+	AluJb	SINE/Alu	1	308	-4	18
2213	11.2	0.3	0.3	17193	17489	C	AluSx	SINE/Alu	-14	298	2	19
434	28.5	4.1	5.7	18517	18713	C	L3	LINE/CR1	-248	3851	3658	20
1282	19.7	5.8	0.7	18743	19033	+	L1MC4a	LINE/L1	7571	7876	-6	21
586	24.5	23.3	1.8	19060	19342	+	MLT2F	LTR/ERVL	1	343	-320	22
727	33.6	2.2	0.6	19497	19816	C	L3	LINE/CR1	-578	3521	3197	20
9033	7.9	0.3	0.8	19831	20999	+	L1P2	LINE/L1	1	1163	-5021	23
5544	8.5	5	0.3	21000	21744	C	LTR17	LTR/ERV1	0	780	1	24
2414	6.3	1.4	0	21745	22044	C	HERV17-int	LTR/ERV1	0	8626	8335	24
291	5	0	0	22023	22062	C	HERV17-int	LTR/ERV1	-1078	7548	7509	24
30151	5	0.4	0.1	22055	25635	C	HERV17-int	LTR/ERV1	-2607	6019	2430	24
7994	5.4	0.4	0.6	25619	26593	C	HERV17-int	LTR/ERV1	-6610	2016	1044	24
2480	6.1	0	1	26596	26909	C	LTR17	LTR/ERV1	-469	311	1	25
9243	6	1.4	0.2	26911	30907	+	L1PA6	LINE/L1	1320	6328	-1	26

293	25	2.3	2.3	30992	31077	C	L3	LINE/CR1	-1030	3069	2984	20
339	29	0	2	31135	31232	C	MamGypLTR3a	LTR/Gypsy	0	981	886	27
1312	20.2	4.8	8.6	31233	31628	C	MLT1B	LTR/ERVL-MaLR	-3	387	6	28
1993	11.6	11	9	31665	31780	C	LTR56	LTR/ERV1	0	445	329	29
1999	12.5	1	1	31781	32071	C	AluSx	SINE/Alu	-21	291	1	30
1993	11.6	11	9	32072	32167	C	LTR56	LTR/ERV1	-117	328	237	29
2155	10.8	2.3	0.6	32168	32474	+	AluSx	SINE/Alu	1	312	0	31
1993	11.6	11	9	32475	32697	C	LTR56	LTR/ERV1	-209	236	1	29
2852	13.2	5	2	32864	33383	C	MER41B	LTR/ERV1	-38	596	1	32
1025	27.2	8	7.2	33652	34039	C	MamGypLTR3a	LTR/Gypsy	-590	391	1	33
402	28.5	17.4	0.7	34115	34361	C	L3	LINE/CR1	-1373	2726	2439	20
279	29.9	5.5	2.4	34402	34603	C	L3	LINE/CR1	-1738	2361	2154	20
278	34.3	1	1	34695	34794	C	L2b	LINE/L2	-18	3357	3258	34
704	15	3.8	0	34971	35103	+	L1MC1	LINE/L1	5292	5429	-915	35
1447	17.6	4.8	5.8	35101	35413	+	L1MC1	LINE/L1	6018	6327	-6	35
401	33	8.4	6.8	35423	35878	C	L2b	LINE/L2	-274	3101	2655	34
269	33.3	13.2	1.7	35963	36280	C	L3	LINE/CR1	-2006	2093	1740	20
695	19	19	0	36325	36519	C	MIR	SINE/MIR	-24	238	7	36
2139	10.6	3.6	0	36630	36930	+	AluSx	SINE/Alu	1	312	0	37
1895	13.8	0.3	4.2	37088	37382	+	AluJb	SINE/Alu	1	284	-28	38
283	19.1	3.1	12.5	37662	37757	+	MER5B	DNA/hAT-Charlie	66	153	-25	39
2272	9.7	0.3	0	38268	38556	C	AluY	SINE/Alu	-21	290	1	40
1186	16.6	1.9	3.9	38989	39195	+	AluJb	SINE/Alu	86	288	-24	41
2391	9.8	0.7	0	39278	39583	C	AluSx	SINE/Alu	-4	308	1	42
238	16.1	8.4	0.6	39642	39715	C	L1MC1	LINE/L1	0	6344	6264	43
647	29.7	4.9	3.2	39713	40077	C	L1ME4a	LINE/L1	-229	5895	5525	44
2372	18.2	3.6	0.8	40125	40596	+	MLT1D	LTR/ERVL-MaLR	21	505	0	45
2326	9.9	0.3	2	40629	40936	C	AluSx	SINE/Alu	-9	303	1	46
251	28.1	2.6	6.4	41318	41431	+	MLT1D	LTR/ERVL-MaLR	476	585	-10	45
305	30.3	1.6	0.8	41599	41721	+	MLT1D	LTR/ERVL-MaLR	170	293	-1094	45
253	30.4	6	1.6	42367	42483	C	MER5A	DNA/hAT-Charlie	-25	164	43	47
827	28.7	2.8	0.4	43065	43319	C	MIR	SINE/MIR	0	262	2	48
354	22.6	3.5	2.8	43399	43539	+	L1MEi	LINE/L1	663	804	-1408	49
441	26.8	7.3	0	43510	43549	+	L1MEi	LINE/L1	1174	1221	-991	49
2075	12.4	1	0	43550	43839	+	AluSx	SINE/Alu	20	312	0	50
441	26.8	7.3	0	43840	43962	+	L1MEi	LINE/L1	1222	1348	-864	49
268	18.8	2.1	0	43974	44021	+	MLT1D	LTR/ERVL-MaLR	1258	1306	-81	45
404	28	7.8	0.5	44655	44854	+	L1ME4c	LINE/L1	5474	5688	-436	51
515	32.8	0.5	0	45402	45587	C	L1ME4a	LINE/L1	-262	5862	5676	52
242	26.4	2.8	7.8	46718	46824	+	L2a	LINE/L2	3109	3210	-177	53
452	24.8	6	0	46906	47038	+	L2a	LINE/L2	3267	3407	-19	53
364	27.5	3.6	0.9	47121	47230	+	SVA_A	Retroposon/SVA	200	312	-1075	54
473	18.2	1.3	0	47174	47250	C	7SLRNA	srpRNA	-241	79	2	55
205	21.7	4.3	0	47640	47685	C	MIR3	SINE/MIR	-55	153	106	56
611	26	1.6	2.7	47897	48082	+	Eutr18	LTR	246	429	-207	57
2270	9.3	0.3	1	48410	48712	+	AluSx	SINE/Alu	1	301	-11	58
859	22.7	19.1	0.9	48865	49280	C	L2	LINE/L2	-421	2998	2508	59

290	24.4	17.7	1.5	49311	49485	C	MIRb	SINE/MIR	-30	238	36	60
258	22.7	38.6	0	50252	50414	C	MIRb	SINE/MIR	-9	253	34	61
341	22.2	1.4	0	51987	52058	C	MIR	SINE/MIR	-129	133	61	62
213	31.5	4.8	9.2	52793	52939	C	L2d	LINE/L2	-37	3388	3248	63
184	32	5	5.7	53657	53815	C	L2d2	LINE/L2	-10	3365	3208	64
293	17.5	0	0	53888	53950	+	L1MD	LINE/L1	5696	5758	-2237	65
285	20.3	10.1	3.4	53998	54108	C	L2d2	LINE/L2	-250	3137	3031	64
255	27.9	9.2	1.1	54114	54200	C	MER117	DNA/hAT-Charlie	-1	196	103	66
221	29.4	9.4	0	54393	54477	C	MIR3	SINE/MIR	-76	132	40	67
368	37.4	6.2	1.8	55675	56091	+	L2c	LINE/L2	1982	2416	-1003	68
424	28.8	6.4	5.5	56509	56943	C	L2c	LINE/L2	-2	3373	2888	69
270	26	10.6	0	57059	57162	C	L2c	LINE/L2	-675	2744	2630	69
1121	12.4	3.8	0.5	57323	57508	C	MER53	DNA/hAT-Blackjack	-1	192	1	70
864	35	11.1	1.6	57910	58215	C	L2c	LINE/L2	-1559	1860	1526	69
2067	11.9	0	2	58216	58523	C	AluSx	SINE/Alu	-9	303	2	71
864	35	11.1	1.6	58524	59214	C	L2c	LINE/L2	-1894	1525	770	69
246	31	2.4	11.9	59234	59398	+	LTR89	LTR/ERVL	556	706	-173	72
237	42	1	2	60022	60218	+	L2c	LINE/L2	3069	3263	-124	68
420	30.8	11.9	1.1	60223	60475	C	L2c	LINE/L2	-2	3385	3106	73
266	32.9	5.2	1.2	60498	60651	+	L2b	LINE/L2	3209	3368	-7	74
279	30.8	5.5	1.8	61858	62012	+	MIRb	SINE/MIR	21	183	-85	75
1097	27.1	10.2	3.9	62076	62735	C	L2a	LINE/L2	-3	3423	2713	76
414	29.9	10.8	2.9	63204	63568	C	L2a	LINE/L2	-27	3399	2968	77
442	34.8	12.1	2.5	63579	64209	+	L2b	LINE/L2	2691	3374	-1	78
1063	24.7	8.5	0.8	66131	66493	+	L2a	LINE/L2	3036	3426	0	79
729	21.6	3.9	0	66555	66707	+	MER58A	DNA/hAT-Charlie	1	159	-65	80
366	34	13.3	4.8	66744	67111	+	L2b	LINE/L2	2977	3374	-13	81
2099	11.5	0	2.7	67114	67416	+	AluSx	SINE/Alu	2	296	-16	82
339	14	0	1.8	67468	67525	+	MER58A	DNA/hAT-Charlie	168	224	0	83
772	23.8	2.8	0	67526	67706	C	MIR	SINE/MIR	-64	198	13	84
1652	16.7	9.7	5.3	68067	68446	C	MSTD	LTR/ERVL-MaLR	0	396	1	85
1807	11.4	6.6	0	68527	68799	+	AluSx	SINE/Alu	4	294	-18	86
229	26.8	28.3	0	69276	69413	C	MIRc	SINE/MIR	-20	248	72	87
408	31.5	12.4	4.1	69841	70245	+	L2c	LINE/L2	2931	3374	-13	88
240	30.1	19.4	1.7	70817	71129	C	L2c	LINE/L2	0	3387	3024	89
248	29.7	8.4	3	71749	71939	+	L2c	LINE/L2	2943	3143	-244	90
324	29.2	7	1.6	71928	72042	+	L2c	LINE/L2	3267	3387	0	90
810	25.4	3.4	0	72231	72466	C	Zaphod5b	DNA/hAT-Tip100	-99	307	64	91
194	14.7	0	0	72571	72604	C	L2d	LINE/L2	-4	3421	3388	92
512	32.4	8.7	1.3	72705	72923	C	MIRb	SINE/MIR	0	268	34	93
437	32	6.2	2.2	72926	73157	C	MIRb	SINE/MIR	-2	266	18	94
861	28.8	0	6.1	73892	73971	C	MIR	SINE/MIR	-30	232	163	95
2389	7.2	0	0	73972	74278	C	AluY	SINE/Alu	-4	307	1	96
861	28.8	0	6.1	74279	74441	C	MIR	SINE/MIR	-100	162	3	95
185	31.8	6.1	0	75673	75738	C	MIRc	SINE/MIR	-76	192	123	97
562	31	7.2	1.2	77268	77502	C	MIRb	SINE/MIR	-6	262	14	98
562	24.4	9.2	1.7	77556	77718	C	MIRb	SINE/MIR	-23	245	71	99

204	26.9	1.9	0	77721	77772	C	MIRb	SINE/MIR	-167	101	49	99
283	21.7	14.4	0	78035	78115	+	L2a	LINE/L2	3348	3432	-32	100
332	30.6	3.2	0.8	78132	78256	C	L2c	LINE/L2	-2	3385	3258	101
431	31.5	3.4	2.9	78308	78513	C	L2c	LINE/L2	-143	3244	3038	101
243	36.2	3.9	2.2	81740	81917	+	L2d	LINE/L2	3077	3257	-168	102
201	20.2	0	0	82778	82826	C	L2c	LINE/L2	-293	3171	3122	103
265	28.9	16.5	3.1	83962	84161	C	L3	LINE/CR1	-2	4097	3872	104
186	16.1	0	0	84667	84697	C	MIRb	SINE/MIR	-23	245	215	105
225	28.8	0	0	84766	84824	C	MIR	SINE/MIR	-177	85	27	106
224	30.5	17.1	1.7	84932	85188	+	L2	LINE/L2	2592	2887	-532	107
197	32.4	8	3.4	85511	85622	C	L2c	LINE/L2	-3	3384	3268	108
229	34.9	2.8	0	86397	86505	C	L2b	LINE/L2	-44	3331	3220	109
747	24.8	2.4	7	86538	86789	C	L2a	LINE/L2	-1	3425	3185	110
1257	29.5	16.3	2.2	86861	87868	C	L2a	LINE/L2	-225	3162	2013	110
1712	17.9	3.3	0	87994	88295	C	AluJb	SINE/Alu	0	312	1	111
318	34.1	0	0.8	88331	88444	C	MER5A	DNA/hAT-Charlie	-2	187	75	112
1632	11	0.4	0	88445	88681	+	AluSx	SINE/Alu	1	238	-74	113
318	34.1	0	0.8	88682	88696	C	MER5A	DNA/hAT-Charlie	-115	74	60	112
1633	9.4	1.2	18.4	89059	89383	C	AluSx	SINE/Alu	-34	278	1	114
315	35.1	2	0	89578	89725	C	L2	LINE/L2	-1153	2266	2116	115
610	33.8	5.6	4.9	89844	90441	+	L2b	LINE/L2	2772	3373	-2	116
401	32	4.4	1.3	94470	94697	C	L3	LINE/CR1	-8	4091	3857	117
886	32.7	6.3	2.5	94707	95315	C	CR1-3_Croc	LINE/CR1	-299	3274	2384	118
344	29.2	8.8	5.7	95334	95703	+	L2d	LINE/L2	3043	3419	-7	119

3. RepeatMasker alignments from the THOLE locus as discussed in the manuscript

THOLE exons have been highlighted red.

A) RepeatMasker alignments of the LTR8/HUERS-P1 region

```
5025 9.56 1.60 0.87 NC_000001.11:109831234-109931057 73 518 (99306) LTR8#LTR/ERV1 1
453 (238) c_b1s401i0 1

  NC_000001.11:         73 TGAAACCGCCTTTGCAAAATTATGACTGAGACAGTGAAAGAGATCTAACC 122
                                                  i                      i
  LTR8#LTR/ERV1          1 TGAAACCGCCTTTGCAAAATTATAACTGAGACAGTGAAAGAGATCTGACC 50

  NC_000001.11:        123 TTACTGACTCCATCTTGCTTCTAGCCTCAAAGCTGCCCTTGTAGG---CT 169
                            v  i                  i    v      i      vvi---
  LTR8#LTR/ERV1         51 TAACCGACTCCATCTTGCTTCTAACCTCCAAGCTGTCCTTGTTCATTCCT 100

  NC_000001.11:        170 GGGTGTACGCTGAACTAATTTGGGGGGAAACTTAGTTTATAGTTTA---- 215
                              i   v  i       i  v   i i                  ----
  LTR8#LTR/ERV1        101 GGGCGTAGGCCGAACTAACTTTGGGAGGAACTTAGTTTATAGTTTANANT 150

  NC_000001.11:        216 ---AATAAAGATGGTAACAGCCCTTTCCCAAAGCAGGCCTCCTTCTTGCC 262
                           ---  i     i i                  i  ii  i
  LTR8#LTR/ERV1        151 TTAAACAAAGACGATAACAGCCCTTTCCCAAAACAAACCCCCTTCTTGCC 200

  NC_000001.11:        263 TGGGGACTAGATTGCCTTTGTAGGACTAACATTACCTGCAAGATTAGAAA 312
                                      i                      v ii
  LTR8#LTR/ERV1        201 TGGGGACTAGACTGCCTTTGTAGGACTAACATTAGCCACAAGATTAGAAA 250

  NC_000001.11:        313 TTATGGTTTAGGAGTCATGCAGCTGGAGACTTCAAGATTCCGATCCTCCC 362
                                                       i  v        i  i
  LTR8#LTR/ERV1        251 TTATGGTTTAGGAGTCATGCAGCTGGAGGCTACAAGATTCTGACCCTCCC 300

  NC_000001.11:        363 TAAACTGCTCCTAAGATCATTGCTTGAGATATTTTGTAGACCCTGCGCTT 412
                           i                  v                i         i
  LTR8#LTR/ERV1        301 CAAACTGCTCCTAAGATCAGTGCTTGAGATATTTTGCAGACCCTGCACTT 350

  NC_000001.11:        413 GATGAATCAGCTGGCACCACCCAGATCAGTAAACTGGTTCATCTGATCTT 462
                               i                      ii        i
  LTR8#LTR/ERV1        351 GATGGATCAGCTGGCACCACCCAGATCGATAAACTGGCTCATCTGATCTT 400

  NC_000001.11:        463 GTAGCCCCCACCCAGGAACTGACTCAGTGCAAGAAGACAGGTCCGACTCC 512
                             i                        i            v i
  LTR8#LTR/ERV1        401 GTGGCCCCCACCCAGGAACTGACTCAGCGCAAGAAGACAGCTTCGACTCC 450

  NC_000001.11:        513 CTATTA 518
                              ---
  LTR8#LTR/ERV1        451 CTA--- 453


2211 11.65 0.97 0.32 NC_000001.11:109831234-109931057 519 828 (98996) C
AluSx#SINE/Alu (0) 312 1 c_b1s352i0 2

  NC_000001.11:        519 TTTCTTTCTTTCTTTCTTTTTTTTTTTTTTTGAGACAGGGTCTCACACTGT 568
                              i   i   i   i                   i i     i v
C AluSx#SINE/Al        312 TTTTTTTTTTTTTTTTTTTTTTTTTTTTTTTGAGACGGAGTCTCGCTCTGT 263

  NC_000001.11:        569 TGGCCAGGCTGGAGTGCAGTGGTGCGATCTTGGCTCACTGCAACCTCCAC 618
                           i v                   i       i                 i
C AluSx#SINE/Al        262 CGCCCAGGCTGGAGTGCAGTGGCGCGATCTCGGCTCACTGCAACCTCCGC 213
```

```
  NC_000001.11:        619 CT-CCATATTCAAGCGATTCTCCTGCCTCAGCCTCCCGAGTAGCTGGGAT 667
                             -  ivi
C AluSx#SINE/Al        212 CTCCCGGGTTCAAGCGATTCTCCTGCCTCAGCCTCCCGAGTAGCTGGGAT 163

  NC_000001.11:        668 TACAGGTGGCTGCCACCACGCCCGGCTAAGTTTTATTATTTTTAATAAAG 717
                                 i vvi                  v    i-        i  i
C AluSx#SINE/Al        162 TACAGGCGCGCGCCACCACGCCCGGCTAATTTTTG-TATTTTTAGTAGAG 114

  NC_000001.11:        718 ATGGGGTTTCACTATGTTGGCCAGGCTGGTCTTGAACTCCTGACCTC--G 765
                            i          i                   i              --
C AluSx#SINE/Al        113 ACGGGGTTTCACCATGTTGGCCAGGCTGGTCTCGAACTCCTGACCTCAGG 64

  NC_000001.11:        766 TGATCCACCTGCCTCAGCCTCCCAAAGTGCTGGGATTATAGGTGTGGGCC 815
                                 i  i     i                      i   i   i
C AluSx#SINE/Al         63 TGATCCGCCCGCCTCGGCCTCCCAAAGTGCTGGGATTACAGGCGTGAGCC 14

  NC_000001.11:        816 ACTGCACCTGGCC 828
                             i  i  i
C AluSx#SINE/Al         13 ACCGCGCCCGGCC 1
```

```
5025 9.56 1.60 0.87 NC_000001.11:109831234-109931057 829 1067 (98757) LTR8#LTR/ERV1
454 691 (0) c_b1s401i0 1

  NC_000001.11:        829 TGTAATTTCATCTCTGACCAATCAGCACTCCTGGCTCACTGGCTTCCCCT 878
                           -- i                           i i               i
  LTR8#LTR/ERV1        454 --TGATTTCATCTCTGACCAATCAGCACTCCCGACTCACTGGCTTCCCCC 501

  NC_000001.11:        879 CACCCACCAAGTTATCCTTAAAAACTCTGCTCCCTGAATGCTCAGGGAGA 928
                           i         i                  v    i        i
  LTR8#LTR/ERV1        502 TACCCACCAAATTATCCTTAAAAACTCTGATCCCCGAATGCTCGGGGAGA 551

  NC_000001.11:        929 CTGATTTGAGTAATAATAAAACTCCCGTCTGCAGCACAG-CAGCTCTGCA 977
                                                    v    v v      - i       i
  LTR8#LTR/ERV1        552 CTGATTTGAGTAATAATAAAACTCCGGTCTCCCGCACAGCCGGCTCTGCG 601

  NC_000001.11:        978 TGAATTACTCTTTCACTGTTGCAATTCCCCTGTCTCAATGAATCAGCTCT 1027
                                         v  i                 ii  i    i
  LTR8#LTR/ERV1        602 TGAATTACTCTTTCTCTATTGCAATTCCCCTGTCTTGATAAATCGGCTCT 651

  NC_000001.11:       1028 GTCTAGGCAGCGGGCAAGGTGAACCCCTTGGGCGGTTACA 1067
                                                     v
  LTR8#LTR/ERV1        652 GTCTAGGCAGCGGGCAAGGTGAACCCGTTGGGCGGTTACA 691
```

```
36461 7.40 2.15 0.57 NC_000001.11:109831234-109931057 1068 2623 (97201) HUERS-
P1#LTR/ERV1 1 1624 (4639) m_b1s701i0 1

  NC_000001.11:       1068 AATTTGGGGGCTCGTCTGGGATTGCCCTTGTGGCTACTTGCCCATGGTTC 1117
                                           i                    i     i
  HUERS-P1#LTR/          1 AATTTGGGGGCTCGTCCGGGATTGCCCTTGTGGCTACCTGCCCGTGGTTC 50

  NC_000001.11:       1118 AGTGGCCTCCTCCCCCAGCGATGAATCCAGAAGTCAGCCCAAGCGGCAGC 1167
                           i  i   i  -- i  i      i       i i             v
  HUERS-P1#LTR/         51 GGTAGCCCCC--CTCCGGCGATGGATCCAGAGGCCAGCCCAAGCGGCCGC 98

  NC_000001.11:       1168 CTGGTTCTCTTGGACTGGGGGCTGACTCTGGTATT-TCTCTACTGGCAGG 1216
                             i                              i -           i
  HUERS-P1#LTR/         99 CTAGTTCTCTTGGACTGGGGGCTGACTCTGGTACTCTCTCTACTGGCGGG 148

  NC_000001.11:       1217 GCACTGCCGACCCGATGTGCATGGATTTAATTGCAGTGGAGAAAGAGTCC 1266
                             i          i                     i        v
  HUERS-P1#LTR/        149 GCGCTGCCGACCCAATGTGCATGGATTTAATTGCAATGGAGAAATAGTCC 198

  NC_000001.11:       1267 TAGGGAGACACCACTTAATTGTAGCCCTATCACAGGGCGTCTGTCTGTAG 1316
                            i       ii v ?   i                  i
  HUERS-P1#LTR/        199 TGGGGAGACGTCCCNTAACTGTAGCCCTATCACAGGGTGTCTGTCTGTAG 248
```

```
NC_000001.11:       1317 CCCCATGGTGGGA----TGTCTGTAGCCCCATTGTGGGCTGTCTGGATTG 1362
                                     i----                 i   v
HUERS-P1#LTR/        249 CCCCATGGTGGGGTGTCTGTCTGTAGCCCCATTGCGGGGTGTCTGGATTG 298

NC_000001.11:       1363 GTAAGTATCCTAGGCACTGCCAATGGCTTCTTCCTTCTCCTGACTGGTTA 1412
                           i            i       i v  i           i        -
HUERS-P1#LTR/        299 GTGAGTATCCTAGGCGCTGCCAACGCCTCCTTCCTTCTCCCGACTGGTT- 347

NC_000001.11:       1413 TGTAGCCCTATGGTGGTGTGTCTGTAGCACCATTGCAGGGTGTCTGTT-- 1460
                                         v           v       i           --
HUERS-P1#LTR/        348 TGTAGCCCTATGGTGGGGTGTCTGTAGCCCCATTGCGGGGTGTCTGTTTG 397

NC_000001.11:       1461 CAGCTC--CTGTGGGG-GTCTCAGT---TGGCTC--TTTC----TAACTA 1498
                         i     -- ii     -    vv  --- i  i --  v ---- iv  i
HUERS-P1#LTR/        398 TAGCTCCACCATGGGGTGTCTGTGTCTGTAGCCCCATTGCGGGGTGTCTG 447

NC_000001.11:       1499 GTCACACAGCTCC--GAAGGT-------------------CTAATAGGAA 1527
                         v --ii       -- ii   -------------------   i
HUERS-P1#LTR/        448 TT--TGCAGCTCCTGGGGGGTCTCGGTTGGCTCTCCCTAACTAGTAGGAA 495

NC_000001.11:       1528 GAGTCCTGGTTTGGGAGACTTGTCTTCAATCAGGAAGATTTCGAGGAGTT 1577
                              i     i         v  i                  i    v
HUERS-P1#LTR/        496 GAGTCTTGGTTCGGGAGACTTCTCCTCAATCAGGAAGATTTCGGGGAGGT 545

NC_000001.11:       1578 TTCTCAGAAGGAGACTAGGAGAATAGTTTGGGAGGGATACTCTTGGAGTT 1627
                                 v     v      i         i
HUERS-P1#LTR/        546 TTCTCAGACGGAGAATAGGAGGATAGTTTGGAAGGGATACTCTTGGAGTT 595

NC_000001.11:       1628 CTTGGTTAGGGATCTGATTTAGAAGGCCTTCT---------GTCTTTGCA 1668
                                             i           ---------       ii
HUERS-P1#LTR/        596 CTTGGTTAGGGATCTGATTTGGAAGGCCTTCTGTCCGTCTCGTCTTTGTG 645

NC_000001.11:       1669 TGTGTTCGTATATTTGGAAGGAATCTCAGAGAGG--TTGCTGATGGAAGT 1716
                               i      v    i  i     ?  -   --
HUERS-P1#LTR/        646 TGTGTTTGTATATGTGGAGGGGATCTCNGA-AGGAATTGCTGATGGAAGT 694

NC_000001.11:       1717 CCAGCAGGCCTAACTCAGAGAACCCTCCTTATTTGTCTGGTCACATTCGA 1766
                                                                          i
HUERS-P1#LTR/        695 CCAGCAGGCCTAACTCAGAGAACCCTCCTTATTTGTCTGGTCACATTCGG 744

NC_000001.11:       1767 TGAGCTCTAAAGAAGGCTCAACAGGCCTGTCTCAGGGGGTGAATATCTGC 1816
                              i  i     i              ?   --       v    i
HUERS-P1#LTR/        745 TGAGCCCTGAAGAAAGCTCAACAGGCCTGNCTC--GGGGTGACTATCCGC 792

NC_000001.11:       1817 TCTTCCCATTGCTCAGATACCCCGTTGTGAATTACCATTTGGAGGTAGTC 1866
                              v v    i    v     i            i  i      vi
HUERS-P1#LTR/        793 TCTTCGCCTTGCCCAGAGACCCCATTGTGAATTACCGTTCGGAGGTCATC 842

NC_000001.11:       1867 CGTCCCCACCTGGAGTGGATCAAAGACAACAGAGGCCAACGGGGAAAAAG 1916
                          v                              i i     -
HUERS-P1#LTR/        843 CCTCCCCACCTGGAGTGGATCAAAGACAACAGGGACCAAC-GGGAAAAAG 891

NC_000001.11:       1917 TTTGAGCTTTGCCAGGCTGATATTGGCTGCTGAATAAGGTGACTAATGTC 1966
                                         ii        v       ii         i
HUERS-P1#LTR/        892 TTTGAGCTTTGCCAGGTCGATATTGGGTGCTGAACGAGGTGACTAGTGTC 941

NC_000001.11:       1967 TGTTTTGTTATGTGTATTTTGCTGGGATGGAAAATGTTAATTTGGTTTCC 2016
                                                                   i    i
HUERS-P1#LTR/        942 TGTTTTGTTATGTGTATTTTGCTGGGATGGAAAATGTTAATTCGGTTCCC 991

NC_000001.11:       2017 CATGCAGCCCGTCAGGCAGCATCTTGCAAATAAAGAATCTTGTC-----T 2061
                                     ii                 vi         i -----
HUERS-P1#LTR/        992 CATGCAGCCCGTTGGGCAGCATCTTGCAAATTGAGAATCTTGCCTATGGT 1041

NC_000001.11:       2062 TAC------------AGGGTGATTTTCTCTTGTAAAATGGCTTGAACCCC 2099
                          v ------------             i       i        ?
HUERS-P1#LTR/       1042 TCCATAAAACAGAAAAGGGTGATTTTCTTTTGTAAAGTGGCTTGANCCCC 1091
```

```
  NC_000001.11:       2100 AAAGCTATAGCACAAGCAAGCAGGGTCATCAGAAGCCACTCCATTCTTCT 2149
                            v      i  i  ?  i                   i    i
  HUERS-P1#LTR/       1092 ACAGCTATGGCGCANGCGAGCAGGGTCATCAGAAGCCGCTCCGTTCTTCT 1141

  NC_000001.11:       2150 GGAAGCTGCAGGGAAATGGAACCCAGAAACCAGGTATGACAGCAAAAAGG 2199
                                      i    v       i      v      v i
  HUERS-P1#LTR/       1142 GGAAGCTGCAGAGAAAGGGAACCCGGAAACCTGGTATGCCGGCAAAAAGG 1191

  NC_000001.11:       2200 GTAAAAAATTCTTACCGGCCAAGTTTCTGA----TCTGTCTTGCTTTCTC 2245
                               i           i            i----   v   iv
  HUERS-P1#LTR/       1192 GTAAGAAATTCTTACCAGCCAAGTTTCTGGTCTCTCTCTCTCTCTTTCTC 1241

  NC_000001.11:       2246 TGTCTGAAT-AAACGGTAAACTATTTGTCTCCTCTGCAAGGGTTTGATTA 2294
                                 ?i -
  HUERS-P1#LTR/       1242 TGTCTGNGTAAAACGGTAAACTATTTGTCTCCTCTGCAAGGGTTTGATTA 1291

  NC_000001.11:       2295 ACAGAAAAAAAGGATTTGTGAGACTAGTCTTAAGCTGTAA-AAATCTGGTG 2343
                            i                             i      i-
  HUERS-P1#LTR/       1292 ATAGAAAAAAAGGATTTGTGAGACTAGTCTTAGGCTGTAGCAAATCTGGTG 1341

  NC_000001.11:       2344 TGCTTTGTGCTAAGAATTTGTCTTTCTGTGTTCTGTAATGGAGAGAGGGG 2393
                            i
  HUERS-P1#LTR/       1342 TACTTTGTGCTAAGAATTTGTCTTTCTGTGTTCTGTAATGGAGAGAGGGG 1391

  NC_000001.11:       2394 TATCACAGGATAGAACATGGGTTTAGGACCCC--TAAGCCTGCTTTTAAA 2441
                                           i               --      i      v
  HUERS-P1#LTR/       1392 TATCACAGGATAGAACGTGGGTTTAGGACCCCTATAAGCCCGCTTTTCAA 1441

  NC_000001.11:       2442 GTCAGCTTGGCAGGCTGGTCAGTTATAAACTTTGCTATGGGTCCCTGAAA 2491
                            i    ii                 i          ii
  HUERS-P1#LTR/       1442 GCCAGCCCGGCAGGCTGGTCAGTTACAAACTTTGCTGCGGGTCCCTGAAA 1491

  NC_000001.11:       2492 CCAATACGGTATGAAATTTCTCTGTTTTGTTTTGTGTCCTTAAGAGTTTA 2541
                                  v v               i                    i
  HUERS-P1#LTR/       1492 CCAATACCGGATGAAATTTCTCTGTCTTGTTTTGTGTCCTTAAGAGCTTA 1541

  NC_000001.11:       2542 ACCTTATGACCATG-GGAGATACTTTCTTTTGGTTTCTACCACCCAGACG 2590
                                i        -  i          i        ii   i     v
  HUERS-P1#LTR/       1542 ACCTTGTGACCATGTGGGGATACTTTCTCTTGGTTTCCGCCATCCAGAGG 1591

  NC_000001.11:       2591 ACAGGAATTTTGGGGTTCATGTCATAATCCTAA-- 2623
                                                     i --  i--
  HUERS-P1#LTR/       1592 ACAGGAATTTTGGGGTTCATGTCATAGT--TAGCC 1624
```

1655 11.74 11.19 1.65 NC_000001.11:109831234-109931057 2624 2900 (96924) C
AluSx#SINE/Alu (9) 303 1 c_b1s352i1 3

```
  NC_000001.11:       2624 TTTTTTTTTTTTTTTTTTTTTGAGATGGAGTCTTGCTCCCATTACGAGGG 2673
                                                    i       i    i-i ii vvi
C AluSx#SINE/Al        303 TTTTTTTTTTTTTTTTTTTTTGAGACGGAGTCTCGCTCT-GTCGCCCAGG 255

  NC_000001.11:       2674 CTGGAGTGCAGTGGTGCAATCTCAGCTGAATGCAACCTCTGCCTCCCGGG 2723
                                         i  i     i   v v         i
C AluSx#SINE/Al        254 CTGGAGTGCAGTGGCGCGATCTCGGCTCACTGCAACCTCCGCCTCCCGGG 205

  NC_000001.11:       2724 -------GGTATTCCTTCCTCAGCCTCCCCAGCAGCTGGGATTACA---- 2762
                           ------- i vi    v            v  i                ----
C AluSx#SINE/Al        204 TTCAAGCGATTCTCCTGCCTCAGCCTCCCGAGTAGCTGGGATTACAGGCG 155

  NC_000001.11:       2763 --------------------ATTTTTGTATTTTTAGTAGAGACAGTGTTT 2792
                           --------------------                       i v
C AluSx#SINE/Al        154 CGCGCCACCACGCCCGGCTAATTTTTGTATTTTTAGTAGAGACGGGGTTT 105

  NC_000001.11:       2793 CACCATGTTGGCCAGGCCGGTATCGAACTCCTGACCTCAAGTGATCCACC 2842
                                            i   v                 i       i
C AluSx#SINE/Al        104 CACCATGTTGGCCAGGCTGGTCTCGAACTCCTGACCTCAGGTGATCCGCC 55
```

```
  NC_000001.11:       2843 CGCCTCAGCCTCCCAAAGTGCTAGGATTACAGACAGGCGTGAGCCACTGT 2892
                                 i               i     ----               i i
C AluSx#SINE/Al         54 CGCCTCGGCCTCCCAAAGTGCTGGGATT----ACAGGCGTGAGCCACCGC 9

  NC_000001.11:       2893 GCCCAGCC 2900
                               i
C AluSx#SINE/Al          8 GCCCGGCC 1
```

36461 7.40 2.15 0.57 NC_000001.11:109831234-109931057 2901 5645 (94179) HUERS-P1#LTR/ERV1 1625 4368 (1895) m_b1s701i0 1

```
  NC_000001.11:       2901 TAAAAATTTTTCTTGAGCAATTAAAAGCC-TTGCAAGCTTGAAATTGGCT 2949
                                   ? ?        i         -
  HUERS-P1#LTR/       1625 TAAAAATTNTNCTTGAGCAGTTAAAAGCCTTTGCAAGCTTGAAATTGGCT 1674

  NC_000001.11:       2950 TCTCTAGGCTCCTTCTGGGAAAAGCAATAGGAACTTCTCAATGCTGTATA 2999
                           ?                             i    v
  HUERS-P1#LTR/       1675 NCTCTAGGCTCCTTCTGGGAAAAGCAATAGAAACTGCTCAATGCTGTATA 1724

  NC_000001.11:       3000 GCTCGGTAGCTAAGGCTTTATCTTTTGACACTGGTGGCCTGGGTTCAATT 3049
                               i              i          v   i    i
  HUERS-P1#LTR/       1725 GCTCAGTAGCTAAGGCTTTGTCTTTTGACAATGGCGGCCCGGGTTCAATT 1774

  NC_000001.11:       3050 GTTGGCTGCTGAAATGATTCATTTCTGGTTTGTTATTTGTGTAACGTTGC 3099
                           v      v i i        v                        v
  HUERS-P1#LTR/       1775 CTTGGCTTCCGGAATGATTCCTTTCTGGTTTGTTATTTGTGTAACTTTGC 1824

  NC_000001.11:       3100 CATTTATTGAGGTTTTTTCCCATCGTAGATAGCTTCTGATTTCCTCTCTT 3149
                                          i     vi i i                  v
  HUERS-P1#LTR/       1825 CATTTATTGAGGTTTCTTCCCCCCATGGATAGCTTCTGATTTCCTGTCTT 1874

  NC_000001.11:       3150 GAATTTTCCTTTCTCTGAACTACCTTGTGGAGATTCTAAATCTTATAAAA 3199
                                                      v                i --
  HUERS-P1#LTR/       1875 GAATTTTCCTTTCTCTGAACTACCTTGGGGAGATTCTAAATCTTGT--AA 1922

  NC_000001.11:       3200 AAAAGAAACTGCTTACCATGTCTTTGAAGCACCTAGGAGGTTACATTTGG 3249
                                              ?              i         v
  HUERS-P1#LTR/       1923 AAAAGAAACTGCTTACCATNTCTTTGAAGCACCTGGGAGGTTACCTTTGG 1972

  NC_000001.11:       3250 TAAAGTTCAGAAGCTAGAAGTATTGGCCACTTGGCATGGCTAAAGTCAGG 3299
                                    i    i    i        i                  i
  HUERS-P1#LTR/       1973 TAAAGTTCAAAAGCCAGAAATATTGGCCGCTTGGCATGGCTAAAGTCGGG 2022

  NC_000001.11:       3300 TAATAAGAGATTTGAAAAGATTTCTTTTTTAAAGAGCACTATGGTTAAAA 3349
                                        i   i                   i
  HUERS-P1#LTR/       2023 TAATAAGAGATTTAAAAGGATTTCTTTTTTAAAGAGCGCTATGGTTAAAA 2072

  NC_000001.11:       3350 GTTAGGTTAATTAAAAGTGGATAAACAAGCTATAGATGTATTTAAAAGGC 3399
                             i  v                 ??          i i
  HUERS-P1#LTR/       2073 GTCAGCTTAATTAAAAGTGGATANNCAAGCTATAGGTATATTTAAAAGGC 2122

  NC_000001.11:       3400 CAT--TGTTTTTCTCTTCTTGGAACTTGTTTTTCTGGAAAAAGGTTTTTC 3447
                            v --                  v                         i
  HUERS-P1#LTR/       2123 CTTTATGTTTTTCTCTTCTTGGATCTTGTTTTTCTGGAAAAAGGTTTTTT 2172

  NC_000001.11:       3448 CTTCTAAGTCGACTGAACTATTTTTCTCCATTTTTTTGTCTTGCCACTCT 3497
                                v           i                  i
  HUERS-P1#LTR/       2173 CTTCTCAGTCGACTGAATTATTTTTCTCCATTTTTTCGTCTTGCCACTCT 2222

  NC_000001.11:       3498 TAATACACACATCAGAGCCCCTAAGATAACTTCTGGTAGCTTGGGACTCC 3547
                               i       v    v                      i
  HUERS-P1#LTR/       2223 TAATGCACACATGAGAGGCCCTAAGATAACTTCTGGTAGCCTGGGACTCC 2272

  NC_000001.11:       3548 TTGGGAAAAACAGAGGAGGTGCTGCAGACCCCGCTTTGGGGAAAAAAACC 3597
                                              i  ii         i      i      ???
  HUERS-P1#LTR/       2273 TTGGGAAAAACAGAGGAGGCGCCACAGACCCCGTTTTGGGAAAAAAANNN 2322
```

```
NC_000001.11:       3598 CAAAACACTCTGTTTTCATCATGAAACCCCAGGAATTAATAGCGGATAGA 3647
                          ------          v     i               v
HUERS-P1#LTR/       2323 C------CTCTGTTTTCCTCATGGAACCCCAGGAATTAAAAGCGGATAGA 2366

NC_000001.11:       3648 TCCCTCTCAAAATCAAAGGCT-TGTTCTGTTTTGCATTGTGTTGTCTGAC 3696
                                       ?      -                     i
HUERS-P1#LTR/       2367 TCCCTCTCAAAATCNAAGGCTCTGTTCTGTTTTGCATTGTGTTATCTGAC 2416

NC_000001.11:       3697 GGTTTTGAGTTTGGGGGGCATCAGAAATTACTTTGCATTATGAGAGAGCT 3746
                                     v     i              i
HUERS-P1#LTR/       2417 GGTTTTGAGTTTTGGGGGTATCAGAAATTACTTCGCATTATGAGAGAGCT 2466

NC_000001.11:       3747 TTGGTGTGTAATAACTAGGTAGGAAATATACTCTAAGGGATGGCTAATAG 3796
                                                         i               i
HUERS-P1#LTR/       2467 TTGGTGTGTAATAACTAGGTAGGAAATATACTTTAAGGGATGGCTAATGG 2516

NC_000001.11:       3797 TAGTTAGGGAGGGGTACTTGACTCTTTGCACACTTGGATCAGAGAAGCAT 3846
                         i     v      i      i          i
HUERS-P1#LTR/       2517 CAGTTATGGAGGGATACTTGGCTCTTTGCACGCTTGGATCAGAGAAGCAT 2566

NC_000001.11:       3847 GCTCTTGGCCACCTGGAAGATAAGGAGACATCCCCACCCCCCACTGGGAG 3896
                                               v   i                   i
HUERS-P1#LTR/       2567 GCTCTTGGCCACCTGGAAGATATGGAAACATCCCCACCCCCCACTGAGAG 2616

NC_000001.11:       3897 ATGAGACTCTCATGAGGGATGGGCTGATTGCAAAATGGGCTGATTGGCTT 3946
                                  i    i              i
HUERS-P1#LTR/       2617 ATGAGACTCCCATGGGGGATGGGCTGATTACAAAATGGGCTGATTGGCTT 2666

NC_000001.11:       3947 TGGGTTGCTTTGCAATGAAATGCAGGGTAGAAGCACTGCACTGTCCTCTC 3996
                                 i               ?                    i
HUERS-P1#LTR/       2667 TGGGTTGCCTTGCAATGAAATGCANGGTAGAAGCACTGCACTGTCTTCTC 2716

NC_000001.11:       3997 CAGTAGTATTTCCCTCCTTTT-GGGATCCAGGAT-CAGTATAAAATGGCA 4044
                          v                   -            - i
HUERS-P1#LTR/       2717 CCGTAGTATTTCCCTCCTTTTGGGGATCCAGGATCCGGTATAAAATGGCA 2766

NC_000001.11:       4045 CCCTTAATTTTGGGGATGTGTCTCTGCCTTCAGCTGCTTATTTGCTGCTT 4094
                                          v     i            ?? ??
HUERS-P1#LTR/       2767 CCCTTAATTTTGGGGATCTGTCTTTGCCTTCAGCTGNNTNNTTGCTGCTT 2816

NC_000001.11:       4095 ATTTGGCCTTAGAAATGCATGCTTTCCAGGCCCTGTTCCTCCAAGGGCTC 4144
                            v    i                  v
HUERS-P1#LTR/       2817 ATTAGGCCCTAGAAATGCATGCTTTCCTGGCCCTGTTCCTCCAAGGGCTC 2866

NC_000001.11:       4145 CACCCCGAAGCCAGTAATCCAATTAAGAAACTGGCAAATGAAAAACCTTA 4194
                              i                                       i
HUERS-P1#LTR/       2867 CACCCTGAAGCCAGTAATCCAATTAAGAAACTGGCAAATGAAAAATCTTA 2916

NC_000001.11:       4195 CAAGTGCCTAATCTTC--TCTCTGTATATTTATATGTGTTGTATGTTTAG 4242
                            ?   iv       --   v   i                       v
HUERS-P1#LTR/       2917 CAANTGCTGAATCTTCTGTCTGTGTGTATTTATATGTGTTGTATGTTTAT 2966

NC_000001.11:       4243 ATATAAAAGAGCTCTGATTAATTGGCTTAGAAAAATAAGCGCTTAAATCA 4292

HUERS-P1#LTR/       2967 ATATAAAAGAGCTCTGATTAATTGGCTTAGAAAAATAAGCGCTTAAATCA 3016

NC_000001.11:       4293 AATATTTTGTCAGAAAAATAGAAATTTTAATGCCTTTTT-TTCTCGTCAT 4341
                                                 i             ?-   v   v i
HUERS-P1#LTR/       3017 AATATTTTGTCAGAAAAATAGAAACTTTAATGCCTTTTNGTTCACGTGAC 3066

NC_000001.11:       4342 TTTAGTAATCTTTTGGAAATAAAGACAGTTTTAAAGATTATTGGTAAAAT 4391

HUERS-P1#LTR/       3067 TTTAGTAATCTTTTGGAAATAAAGACAGTTTTAAAGATTATTGGTAAAAT 3116

NC_000001.11:       4392 AAAATGTCTTGAAAATGTAGACATTTGGTCTAAATTAAGATCAGGTATCA 4441
                                   v     ?                      i    i
HUERS-P1#LTR/       3117 AAAATGTCTTCAAAATNTAGACATTTGGTCTAAATTAAGGTCAGATATCA 3166
```

```
NC_000001.11:        4442 GACTTGCTAAATATTTTAAGTTCAAACTGTTTCTTTGACTTTTGAAAATT 4491
                           ii         ii      v        i
HUERS-P1#LTR/        3167 GGTTTGCTAAATGCTTTAAGGTCAAACTGCTTCTTTGACTTTTGAAAATT 3216

NC_000001.11:        4492 GTTCGATTTACCTACTTTGGAGCATTAGATTATAGATAATTCCTGCGGAC 4541
                              i                          v       vv     v
HUERS-P1#LTR/        3217 GTTCAATTTACCTACTTTGGAGCATTAGATTCTAGATAAGGCCTGGGGAC 3266

NC_000001.11:        4542 ATGGGGAGAGCCATGTCCGCTAGCTATGCTAAAAGGAGTCAGACCTTATC 4591
                             v           i  v           i    i
HUERS-P1#LTR/        3267 ATGTGGAGAGCCATGCCCCCTAGCTATGCTGAAAAGAGTCAGACCTTATC 3316

NC_000001.11:        4592 TTCATTTCTGTCTGATGTCCTAGGCTCCACCCCTAGTACATAATTAAAAT 4641
                           ?  i                         ?
HUERS-P1#LTR/        3317 TNCACTTCTGTCTGATGTCCTAGGCTCCACNCCTAGTACATAATTAAAAT 3366

NC_000001.11:        4642 CGCTTACTTGTCAGGTTTTTCACTAAAAATAAAAGGTGCTAAGAGTTAAC 4691
                                   i             i           v
HUERS-P1#LTR/        3367 CGCTTACTTATCAGGTTTTTCACCAAAAATAAAAGTTGCTAAGAGTTAAC 3416

NC_000001.11:        4692 ATTGCAACATGTAGTCGAGACCGCTGGAGAAACAGTTTTACATACAAGGT 4741
                              i        i i     ii
HUERS-P1#LTR/        3417 ATTGTAACATGTAATTGAGACTACTGGAGAAACAGTTTTACATACAAGGT 3466

NC_000001.11:        4742 GTGTAGGGAATGTGTTTTAGGTAAAAGATTATAAGAAGGCATGGGAATAT 4791
                                            v
HUERS-P1#LTR/        3467 GTGTAGGGAATGTGTTTTTGGTAAAAGATTATAAGAAGGCATGGGAATAT 3516

NC_000001.11:        4792 GGCTTTTGTTAAAGGGAATGTAATTTTGTCTAGTTCAGAGGGTTTTAAAG 4841

HUERS-P1#LTR/        3517 GGCTTTTGTTAAAGGGAATGTAATTTTGTCTAGTTCAGAGGGTTTTAAAG 3566

NC_000001.11:        4842 ATTGTCTTAACCTAAAAGAGTAATGGAACAAAACTGAAGGTTTAAGCAAA 4891
                                                    i                      i
HUERS-P1#LTR/        3567 ATTGTCTTAACCTAAAAGAGTAATGGGACAAAACTGAAGGTTTAAGCAAG 3616

NC_000001.11:        4892 GTGAAAAGGGTTTGAAAAGGGTTGATCTTGT---AAAACTTCTGTGGGTA 4938
                          ?             vi               ---    v
HUERS-P1#LTR/        3617 NTGAAAAGGGTTTGTGAAGGGTTGATCTTGTAAAAAAAGTTCTGTGGGTA 3666

NC_000001.11:        4939 TAAACAAGTTGGCTAAGATTTAAAAGAAATTATTTAGC-TTTTCCCCATA 4987
                           i                   i  i ii          -    ii  i
HUERS-P1#LTR/        3667 TGAACAAGTTGGCTAAGATTTGAAGGGGATTATTTAGCTTTTTTTCCGTA 3716

NC_000001.11:        4988 GGTTAAAACATTAAAATCATACTGATGTGGGGCCAGAATCTGGGCCCATG 5037
                                             i       i
HUERS-P1#LTR/        3717 GGTTAAAACATTAAAATCACACTGATGCGGGGCCAGAATCTGGGCCCATG 3766

NC_000001.11:        5038 TGTACGAATAACAGGGTTTTCTTAGAAAATTGATCTGCTGTTTGATGGAA 5087
                             v                                       i i
HUERS-P1#LTR/        3767 TGTCCGAATAACAGGGTTTTCTTAGAAAATTGATCTGCTGTTTAACGGAA 3816

NC_000001.11:        5088 AATTGTAAAGGGTTCTAAAAAGTTTATGAAAATCTTGCCTTATGGTGAAA 5137
                                                              i         v
HUERS-P1#LTR/        3817 AATTGTAAAGGGTTCTAAAAAGTTTATGAAAATCTTACCTTATGGTCAAA 3866

NC_000001.11:        5138 CTAATTAAAACTGGATAGAGATATAAAATTTTATCTAAAAAACTAGCTTT 5187
                                             vv             i -
HUERS-P1#LTR/        3867 CTAATTAAAACTGGATAGATTTATAAAATTTTATTT-AAAAACTAGCTTT 3915

NC_000001.11:        5188 AACGTTAAAAGATGCACTAATGCAAACAAGAAATTTGGTTTTCTCTTTTG 5237
                             i  -                     v
HUERS-P1#LTR/        3916 AACATT-AAAGATGCACTAATGCAAACATGAAATTTGGTTTTCTCTTTTG 3964

NC_000001.11:        5238 AAGATGATTTTTATGTAATGTTAAAAGATAATGAAAGGGTTTTGTTTTCT 5287
                              ?              i                  ?          i
HUERS-P1#LTR/        3965 AAGANGATTTTTATGTAATATTAAAAGATAATGAAAGGNTTTTGTTTTCC 4014
```

```
  NC_000001.11:       5288 CCTTTGGGTAAATGGCAGGGAAAAAAAAGAGGAGAGAGATAAGAGACAGA 5337
                                       i             ii           v
  HUERS-P1#LTR/       4015 CCTTTGGGTAAACGGCAGGGAAAAAAGGGAGGAGAGAGAGAAGAGACAGA 4064

  NC_000001.11:       5338 TTGAGTTGGCCTCATGCTATCTTCATTGGGTCTTGTTTGGAAAGCTAAGT 5387
                             v
  HUERS-P1#LTR/       4065 TTCAGTTGGCCTCATGCTATCTTCATTGGGTCTTGTTTGGAAAGCTAAGT 4114

  NC_000001.11:       5388 CTTCTCTATCAGAGTGAAGGTTTTTCTTTTTTAAAAATTGTTTTGGAGTT 5437
                             i            i                     ?vv
  HUERS-P1#LTR/       4115 CTCCTCTATCAGAGTAAAGGTTTTTCTTTTTTAAAAANATTTTTGGAGTT 4164

  NC_000001.11:       5438 ATCAATTCGGCCAAATGAATGATTTACGGTGATCTGGGA-TCTATTTTGT 5486
                               v  i              i   i     i      -
  HUERS-P1#LTR/       4165 ATCATTTTGGCCAAATGAATGACTTATGGTGACCTGGGATTCTATTTTGT 4214

  NC_000001.11:       5487 GATATCCTGTGTTTTAAACCTTTGATATTTGACAAGCTTTCCAATATCAA 5536
                                  v                           i        v
  HUERS-P1#LTR/       4215 GATATCCAGTGTTTTAAACCTTTGATATTTGACAAACTTTCCAAAATCAA 4264

  NC_000001.11:       5537 ATTATAAATTCTGTCTCTTTCTAACCTAATATTTTAGATATTAGGTTCTT 5586
                                     v           i       ?               i  i
  HUERS-P1#LTR/       4265 ATTATAAATTATGTCTCTTTCTGACCTAATNTTTTAGATATTAGGTCCTC 4314

  NC_000001.11:       5587 TAAAGTCCAAAAATGACATTTGGCTTATTTGGTACAAAAATCACACAGGA 5636
                                                             i        i
  HUERS-P1#LTR/       4315 TAAAGTCCAAAAATGACATTTGGCTTATTTGGTATAAAAATCATACAGGA 4364

  NC_000001.11:       5637 AGCATTGTC 5645
                               -----
  HUERS-P1#LTR/       4365 AGCA----- 4368
```

3466 4.17 7.41 0.00 NC_000001.11:109831234-109931057 5646 6077 (93747) C
LTR7B#LTR/ERV1 (0) 464 1 c_b1s401i1 4

```
  NC_000001.11:       5646 TGTCTCATGCGTCCATGTGAACAGACCACCAAACAGGCTTTGTGTGAGCA 5695
                               v ii      i      v
C LTR7B#LTR/ERV        464 TGTCACGCGCGTCCGTGTGAAGAGACCACCAAACAGGCTTTGTGTGAGCA 415

  NC_000001.11:       5696 ACAAGGCTGTTTATTTCACCTGGGCGCAGGCGGTCTGAGTCTGAAAAGAG 5745
                                                   i        v       i
C LTR7B#LTR/ERV        414 ACAAGGCTGTTTATTTCACCTGGGTGCAGGCGGGCTGAGTCCGAAAAGAG 365

  NC_000001.11:       5746 AGTCAGCGAAGGGTGGTGGGATTATCGTTAGTTCTTATAGGTTTTGGGAT 5795
                                  i                  i
C LTR7B#LTR/ERV        364 AGTCAGCAAAGGGTGGTGGGATTATCATTAGTTCTTATAGGTTTTGGGAT 315

  NC_000001.11:       5796 AGGCGGTGGAGTTAGGAGCAATGTTTTTCGGGCAGGGGGTGGATCTCACA 5845
                                                      v
C LTR7B#LTR/ERV        314 AGGCGGTGGAGTTAGGAGCAATGTTTTGCGGGCAGGGGGTGGATCTCACA 265

  NC_000001.11:       5846 AAGTAAGTTCTCAAGGGTGGGGAGAATTACAAAGAACCTTCCTAA----- 5890
                                vi                                  i   -----
C LTR7B#LTR/ERV        264 AAGTACATTCTCAAGGGTGGGGAGAATTACAAAGAACCTTCTTAAGGGTG 215

  NC_000001.11:       5891 ---------------------------GGTGGGGGAAATTACAAAGTACG 5913
                           ---------------------------         i            i
C LTR7B#LTR/ERV        214 GGGGAGATTACAAAGNACCTTCTTAAGGGTGGGGGAGATTACAAAGTACA 165

  NC_000001.11:       5914 TTGATCACTTAGGGTGGGGCAGAAACAAATCACAATGGTGGAATGTCATC 5963
                                  v
C LTR7B#LTR/ERV        164 TTGATCAGTTAGGGTGGGGCAGAAACAAATCACAATGGTGGAATGTCATC 115

  NC_000001.11:       5964 AGTTAAGGCTATTTTCACTTCTTTTGTGGATCTTCAGTTGCTTCAGGCCA 6013

C LTR7B#LTR/ERV        114 AGTTAAGGCTATTTTCACTTCTTTTGTGGATCTTCAGTTGCTTCAGGCCA 65
```

```
  NC_000001.11:       6014 TCTGGATGTATACGTGCAGGTCACAGGGCATATGATGGCTTAGCTTGGGC 6063
                                                       v
C LTR7B#LTR/ERV         64 TCTGGATGTATACGTGCAGGTCACAGGGGATATGATGGCTTAGCTTGGGC 15

  NC_000001.11:       6064 TCAGAGGCCTGACA 6077

C LTR7B#LTR/ERV         14 TCAGAGGCCTGACA 1


36461 7.40 2.15 0.57 NC_000001.11:109831234-109931057 6078 6650 (93174) HUERS-
P1#LTR/ERV1 4369 4952 (1311) m_b1s701i0 1

  NC_000001.11:       6078 TTGTCAAATATGAAATGGTGTTTGGCTTTCTTTGGTCTCTATTTGTGTAA 6127
                                                              v  v
  HUERS-P1#LTR/       4369 TTGTCAAATATGAAATGGTGTTTGGCTTTCTTTGGGCTATATTTGTGTAA 4418

  NC_000001.11:       6128 ATGTGTTATTGGTATATGTTCTGAAGTAATGTAAAACTCCTATAATTCTA 6177
                                                ii  i v
  HUERS-P1#LTR/       4419 ATGTGTTATTGGTATATGTTCCAAAATTATGTAAAACTCCTATAATTCTA 4468

  NC_000001.11:       6178 ATATGACTTAGTCTATGTTATCTGTAATAATTATAATTATTATGTTAACA 6227
                                       v         v                         vv
  HUERS-P1#LTR/       4469 ATATGACTTAGTATATGTTATCAGTAATAATTATAATTATTATGTTAAAT 4518

  NC_000001.11:       6228 GACTGTGTGCTACAAAGGT---AAATTTCCTTGTCAATTGTGTCTTTAAC 6274
                           ?         i   i    ---
  HUERS-P1#LTR/       4519 NACTGTGTGCCACAGAGGTAACAAATTTCCTTGTCAATTGTGTCTTTAAC 4568

  NC_000001.11:       6275 TGTGGCTGCCCTAAAATGTTTTTGTCATCCACAGATAATTGCTGTCTCGT 6324
                                                              i     i
  HUERS-P1#LTR/       4569 TGTGGCTGCCCTAAAATGTTTTTGTCATCCACAGACAATTGTTGTCTCGT 4618

  NC_000001.11:       6325 TTTGGTCCTCTTTAAAAGATGGTTTTATAATCAGTTTTAAAATGTAACAG 6374
                                                             i v      v
  HUERS-P1#LTR/       4619 TTTGGTCCTCTTTAAAAGATGGTTTTATAATCAGCTATAAAATTTAACAG 4668

  NC_000001.11:       6375 GTGCTCTTAAATGCAGGATTCTGATAAATAACTCTGGAGATTGTGACATT 6424
                                            v       v       -
  HUERS-P1#LTR/       4669 GTGCTCTTAAATGCAGGTTTCTGATTAATAACT-TGGAGATTGTGACATT 4717

  NC_000001.11:       6425 TGAATAGAGG-AAAAACTTTCAAATAGAAGAGTGAAGGGTGTTTGGTTCA 6473
                           v         -                         v           iv
  HUERS-P1#LTR/       4718 AGAATAGAGGAAAAAACTTTCAAATAGAAGAGTGAATGGTGTTTGGTTTT 4767

  NC_000001.11:       6474 CTTTGGACTGTATTTGTATAAATATATTATTAGTATGTATTCCAAAATTA 6523
                                 i                  i            i
  HUERS-P1#LTR/       4768 CTTTGGGCTGTATTTGTATAAATATGTTATTAGTATGTGTTCCAAAATTA 4817

  NC_000001.11:       6524 TGGGAAACTTCTACAATGTTGATATAATTTAGTGTACATTGATAATAAT- 6572
                                    i   i   vi      i           i  iv       -
  HUERS-P1#LTR/       4818 TGGGAAACTCCTATAATTCTGATATGATTTAGTGTACGTTATTAATAATT 4867

  NC_000001.11:       6573 ---ATTGTTATGTAAAA---TTGTATGCCATAGAAGTAATCAAAATTCCT 6616
                           ---              ---          i        i
  HUERS-P1#LTR/       4868 ATAATTGTTATGTAAAATTGTTGTATGCCACAGAAGTAACCAAAATTCCT 4917

  NC_000001.11:       6617 AGTCAATTGTAGCTTTAATAG-GGCTTTAAAATTT 6650
                                                -    v  i v
  HUERS-P1#LTR/       4918 AGTCAATTGTAGCTTTAATAGTGGCTATAGACTTT 4952



8780 9.36 0.98 1.48 NC_000001.11:109831234-109931057 6640 7865 (91959) HUERS-
P1#LTR/ERV1 5044 6263 (0) m_b1s701i1 1

  NC_000001.11:       6640 CTTTAAAATTTGTGCTATTGGAATAGAGAAAAAAACCAAACTTCTAGGAC 6689
                                   v           ----------------
  HUERS-P1#LTR/       5044 CTTTAAAAATTGTGCTATTG----------------CAAACTTCTAGGAC 5077
```

```
NC_000001.11:       6690 TGTCATGGAGAGCTAATGTGTTAAACATTGCTAAACATTTTGTTTCCAGA 6739
                          v            i                   v v        i
HUERS-P1#LTR/       5078 TCTCATGGAGAGCTGATGTGTTAAACATTGCTAATCCTTTTGTTTTCAGA 5127

NC_000001.11:       6740 GTCAAGGGAACTTATTTCTTTAGAGGTATTTGCAACTTTTAACAAATGAA 6789
                               ii              ?   v                   ?   i
HUERS-P1#LTR/       5128 GTCAAGAAAACTTATTTCTTTNGAGCTATTTGCAACTTTTAACAANTGAG 5177

NC_000001.11:       6790 TAAGATATACTCCTGAGAACAAAATTTGGAGCATATTTGTTCCTCTCTAC 6839
                            i           v                         i
HUERS-P1#LTR/       5178 TAAAATATACTCCTGTGAACAAAATTTGGAGCATATTTGTTTCTCTCTAC 5227

NC_000001.11:       6840 CTGATTTCTCCAGAATTTGGAAATTATTTGTGAATATTCTCAATTTATGG 6889
                                                i         i         i
HUERS-P1#LTR/       5228 CTGATTTCTCCAGAATTTGGAAACTATTTGTGAGTATTCTCAACTTATGG 5277

NC_000001.11:       6890 CAGTATAGTTAATTGTGTAAGTGCAATAAGAAGCTATTTTCTTCTGTAAC 6939
                                    v   ii                v  i       i
HUERS-P1#LTR/       5278 CAGTATAGTTATTTGCATAAGTGCAATAAGAATCTGTTTTCTTTTGTAAC 5327

NC_000001.11:       6940 AGAACACAGTTGGAGAAACTGATTATTTTACCAAGGTTTTGAATGGAATG 6989
                           i     i            i              i     v
HUERS-P1#LTR/       5328 AGGACACAATTGGAGAAACTGGTTATTTTACCAAGGCTTTGACTGGAATG 5377

NC_000001.11:       6990 GCATGCTTCCTTTAAAGAATCAAAGTTGACTTATAGAGCCAATTAAAGCC 7039
                           i            i                           v
HUERS-P1#LTR/       5378 GCGTGCTTCCTTTAAGGAATCAAAGTTGACTTATAGAGCCAATAAAAGCC 5427

NC_000001.11:       7040 TGTTGGGGAATCTGACCTCATACCTTGTCTGCACAGTGTCCCCGTACAAG 7089
                         iv     i  ?   i               i     ?     i     i
HUERS-P1#LTR/       5428 CCTTGGGAAANCTGGCCTCATACCTTGTCTACACAGNGTCCCTGTACAGG 5477

NC_000001.11:       7090 GTTCCTGACCTGTGGTAAGTAAGGAATTTCACTTTCTAACAGGCCCAGGA 7139
                                               i    v
HUERS-P1#LTR/       5478 GTTCCTGACCTGTGGTAAGTAAAGAATGTCACTTTCTAACAGGCCCAGGA 5527

NC_000001.11:       7140 ACCCCAAGTTGTCTTGGGACCTCAAGAGGAGAGGAATTTGCCCAACTCAT 7189
                         i         i                            i
HUERS-P1#LTR/       5528 GCCCCAAGTTATCTTGGGACCTCAAGAGGAGAGGAATTTACCCAACTCAT 5577

NC_000001.11:       7190 AGGTATTTGTCGGTACAAACCCACAGCTGGGCTTGGCTTTTAAAAAGTCT 7239
                                  vv            ii        i      ?
HUERS-P1#LTR/       5578 AGGTATTTGAGGGTACAAACCCATGGCTGGGCTCGGCTTTNAAAAAGTCT 5627

NC_000001.11:       7240 TATCTGAGATTCTTCATGGAACAGAGTTCTATCAAAGCCAATTAAAAAAG 7289
                                     i                i             v
HUERS-P1#LTR/       5628 TATCTGAGATTCCTCATGGAACAGAGTTCCATCAAAGCCAATTTAAAAAG 5677

NC_000001.11:       7290 TCTACATGAAAAATAATTATTCTTGCTGTACTTTTTACAAATAATTAGGC 7339
                         i   ?i                      i     v i        i
HUERS-P1#LTR/       5678 CCTANGTGAAAAATAATTATTCTTGCTGCACTTTATGCAAATAATCAGGC 5727

NC_000001.11:       7340 CAATTACAGTAAGACTAAAGTTTATTTTGTAAACGAATCAGTTCTATCAT 7389
                            v                              i
HUERS-P1#LTR/       5728 CAAGTACAGTAAGACTAAAGTTTATTTTGTAAACAAATCAGTTCTATCAT 5777

NC_000001.11:       7390 GATTTGTTTTTAA-AAAAATGAAGACTGGAGAGAGAAAATTTATGCTTCA 7438
                                      -       ii                v
HUERS-P1#LTR/       5778 GATTTGTTTTTAATAAAAATGGGGACTGGAGAGAGAAAAATTATGCTTCA 5827

NC_000001.11:       7439 AAAGAAAAACTATAGTACA-CTGTTGTTAGCTATTCTTGAGGTTTTTTTC 7487
                                            -            i       - ?
HUERS-P1#LTR/       5828 AAAGAAAAACTATAGTACACCTGTTGTTAGCTGTTCTTGA-GNTTTTTTC 5876

NC_000001.11:       7488 TGTAGTTTAGACTAAATTCCAAATTCTTTGTGGGCTAGAAGTCCTCAAAC 7537
                           i     i        i i                 v      i
HUERS-P1#LTR/       5877 TGCAGTTTGGACTAAATCCTAAATTCTTTGTGGGCTACAAGTCCCCAAAC 5926
```

```
  NC_000001.11:       7538 TAATGCTTTCAAATCTTTGTTTTAAAATTGGGAATTGTACTCCTCAGCC 7587
                                             ii        i         i      i v
  HUERS-P1#LTR/       5927 TAATGCTTTCAAATCTTTACTTTTAAAACTGGGAATTGCACTCCTTATCC 5976

  NC_000001.11:       7588 TAGGACTCATTATTTACCTTATAATAGGCTGTTTCACTTAAACACTGTAG 7637
                              ?                   i  v    -   ?      ii     v
  HUERS-P1#LTR/       5977 TAGNACTCATTATTTACCTTATAGTACGCTG-TTCNCTTAAATGCTGTAC 6025

  NC_000001.11:       7638 TAAAACTATAGTTGAGAGTACTAATGTTTTTGCCATGAAAG--------- 7678
                                      v   ? i      i ii         v   ---------
  HUERS-P1#LTR/       6026 TAAAACTATAGATGANAATACTAACGCCTTTGCCATGCAAGCCTTGGAAC 6075

  NC_000001.11:       7679 CCCAGCCAGGCCTGCATGAGT-CACTCAGACAGTTACAAAGCAGTTCAAC 7727
                                                - i           i      i    v
  HUERS-P1#LTR/       6076 CCCAGCCAGGCCTGCATGAGTACGCTCAGACAGTTGCAAAGCGGTTCCAC 6125

  NC_000001.11:       7728 TCTTCTCACCTTGGGATGCACTCCCATTTCCACTACGTCCTCTGTTGGCA 7777
                             i            i vv  ?  i iii        i  i    ii
  HUERS-P1#LTR/       6126 TCCTCTCACCTTGGGGTCAACNCCTACCCCCACTACGCCCCCTGTCAGCA 6175

  NC_000001.11:       7778 GGAAGAAGCCAGAGCGATTTACAACCTTTTCCCATCTTCATAGCCTACAT 7827
                                             iv? ii                     ?   i
  HUERS-P1#LTR/       6176 GGAAGAAGCCAGAGCGATCGNCGGCCTTTTCCCATCTTCATAGCCNACAC 6225

  NC_000001.11:       7828 CGTAAGACGAGGGCATTATAAAACCCAAAGGGAAGGAT 7865
                            v     iv i  ii                  i
  HUERS-P1#LTR/       6226 CTTAAGATTAAGGTGTTATAAAACCCAAAGGGAGGGAT 6263


4851 9.63 4.06 0.14 NC_000001.11:109831234-109931057 7866 8530 (91294)
LTR8#LTR/ERV1 1 691 (0) c_b1s401i2 1

  NC_000001.11:       7866 TGAAACTGCCTTTGCAATATTATGACTGAGACAGTGAAAGAGATCTAAGT 7915
                                 i          v     i                      i vi
  LTR8#LTR/ERV1          1 TGAAACCGCCTTTGCAAAATTATAACTGAGACAGTGAAAGAGATCTGACC 50

  NC_000001.11:       7916 ATACTGACTCCATCTTGTTTCTAACCTCCAAGCTGTCCTTGTAGG---CT 7962
                           vv  i            i                        vvi---
  LTR8#LTR/ERV1         51 TAACCGACTCCATCTTGCTTCTAACCTCCAAGCTGTCCTTGTTCATTCCT 100

  NC_000001.11:       7963 GGGCATAGACTGAACTAACTTTGGGAAAAAACTTAGTTTATAGTT----- 8007
                               i   i i               ii  -              -----
  LTR8#LTR/ERV1        101 GGGCGTAGGCCGAACTAACTTTGGGAGGAA-CTTAGTTTATAGTTTANAN 149

  NC_000001.11:       8008 --TAAACAAAGATGGTAACAGCCCTTTCCCAAAGCAGGCCTCCTTCTTGC 8055
                           --          i i                  i  ii  i
  LTR8#LTR/ERV1        150 TTTAAACAAAGACGATAACAGCCCTTTCCCAAAACAAACCCCCTTCTTGC 199

  NC_000001.11:       8056 ATGGGAACTAGATTGCCTTTGTAGGACTAACATTAGCTGCAAGATTAGAA 8105
                           v    i      i                        ii
  LTR8#LTR/ERV1        200 CTGGGGACTAGACTGCCTTTGTAGGACTAACATTAGCCACAAGATTAGAA 249

  NC_000001.11:       8106 ATTATGGTTTAGGAGTCATGCAGCTGGAGGCTACAAGATTCTGAGCCTCC 8155
                                                                       v
  LTR8#LTR/ERV1        250 ATTATGGTTTAGGAGTCATGCAGCTGGAGGCTACAAGATTCTGACCCTCC 299

  NC_000001.11:       8156 CTAAATAGCTTCCAAGATCAGAGCTTGAGATATTTTGCAGACCCTGCACT 8205
                            i   iv   i i        v
  LTR8#LTR/ERV1        300 CCAAACTGCTCCTAAGATCAGTGCTTGAGATATTTTGCAGACCCTGCACT 349

  NC_000001.11:       8206 TAATGGATCAGCTGGCACCACTAAGATCAATAAACTGGCTCATCTGATCT 8255
                            i                   iv     i
  LTR8#LTR/ERV1        350 TGATGGATCAGCTGGCACCACCCAGATCGATAAACTGGCTCATCTGATCT 399

  NC_000001.11:       8256 TGTGACTCCCACCCAGGAACTGACTCAGTGCAAGAAGACAGGTCCGACTC 8305
                               i i                     i            v i
  LTR8#LTR/ERV1        400 TGTGGCCCCCACCCAGGAACTGACTCAGCGCAAGAAGACAGCTTCGACTC 449
```

```
  NC_000001.11:       8306 CCTATGATTTCATCTCTGACCAATCAGCACTCCTGGCTCACTGGCTT--- 8352
                                                            i i           ---
  LTR8#LTR/ERV1        450 CCTATGATTTCATCTCTGACCAATCAGCACTCCCGACTCACTGGCTTCCC 499

  NC_000001.11:       8353 -----------AGTTATCTTTAAAAACTCTGCTCCCCAAATGCTCAGGGA 8391
                           ----------- i     i            v     i       i
  LTR8#LTR/ERV1        500 CCTACCCACCAAATTATCCTTAAAAACTCTGATCCCCGAATGCTCGGGGA 549

  NC_000001.11:       8392 GACTGATTTGAGTAATAATAAAACTCTGGTCTCCCACATAGCTGGCTCTG 8441
                                                     i        i  i   i
  LTR8#LTR/ERV1        550 GACTGATTTGAGTAATAATAAAACTCCGGTCTCCCGCACAGCCGGCTCTG 599

  NC_000001.11:       8442 CGTGAATTACTCTTTCTCTATTGCAATTCACCTGTCTTGATGAATCGGCT 8491
                                                        v           i
  LTR8#LTR/ERV1        600 CGTGAATTACTCTTTCTCTATTGCAATTCCCCTGTCTTGATAAATCGGCT 649

  NC_000001.11:       8492 CTGTCTAGGCAGCAGGCAAAGCAA---CTTTGGGTGGTTACA 8530
                                        i     i ii --- v     i
  LTR8#LTR/ERV1        650 CTGTCTAGGCAGCGGGCAAGGTGAACCCGTTGGGCGGTTACA 691
```

B) RepeatMasker alignment between the L2 elements and the third THOLE exon.

```
332 30.64 3.20 0.78 NC_000001.11:109831234-109931057 78132 78256 (21568) C
L2c_3end#LINE/L2 (2) 476 349 m_b2s601i50 101

  NC_000001.11:      78132 TCATTCAACTCAGCCATCATTAACTTGGTACCTATTATGTGCTAGGTACC 78181
                                  -i   i v v    v i vi ii     i       i   ii i
C L2c_3end#LINE          476 TCATTCA-TTCAACAAACATTTATTGAGCGCCTACTATGTGCCAGGCGCT 428

  NC_000001.11:      78182 ATTGATCCAGAGGTGACTGAG-CATGGGTGCCACCTGGAAGAAG-TCTCG 78229
                           ivv   v  i  i   v i  -  i  viv ii  ivv   i  -  v i
C L2c_3end#LINE          427 GGGGATACAAAGATGAATAAGACACGGTCCCTGCCCTCAAGGAGCTCACA 378

  NC_000001.11:      78230 GTCTAG-GGTGGGGACAGA-ATGTAACCA 78256
                                 -  v  i      - ?    v
C L2c_3end#LINE          377 GTCTAGTGGAGGAGACAGACANGTAAACA 349


431 31.49 3.40 2.90 NC_000001.11:109831234-109931057 78308 78513 (21311) C
L2c_3end#LINE/L2 (143) 335 129 m_b2s601i51 101

  NC_000001.11:      78308 CAAATGACTTATCAGAGCCAGCATTGAGGAATGAGCCAAGTCCAATGGGA 78357
                              i v v v   v  v  i vv  v ii v  iii vii  v v
C L2c_3end#LINE          335 CAAGTTAATGATAAGTGCTATAATGGGAGTATAGACAGGGTGCTATGGGA 286

  NC_000001.11:      78358 ACACAGGAAAAGGACCCTTTGCTCTGCCAGGAGAGTCACAGAAGGCTTTG 78407
                           i     iii i  vv   vvi  vv? iv  i i    vi        i-
C L2c_3end#LINE          285 GCACAGAGGAGGGCACCTAGACTAGNCTTGGGGGGTCAGGGAAGGCTTC- 237

  NC_000001.11:      78408 CTGGTGGAGGATGAGATGCAATTTCAGCCTGAAATGATGAGGAGTTTGTC 78457
                            i  v     -   v  vii vi v  ?  vi  v      v  --- vv
C L2c_3end#LINE          236 CCGGAGGAGG-TGACATCTGAGCTGAGNCTTGAAGGATGAGTAG---GAG 191

  NC_000001.11:      78458 TTCAGCCATGTGGAGAAAGG-------AGCATTCCAGGCAGAGGGACCAG 78500
                             -     v     iii   -------                   v
C L2c_3end#LINE          190 TT-AGCCAGGTGGAAGGAGGGGGGAAGAGCATTCCAGGCAGAGGGAACAG 142

  NC_000001.11:      78501 TCTGAGCAAAGGC 78513
                           iv  v
C L2c_3end#LINE          141 CATGTGCAAAGGC 129
```

4. Supplementary Figure 1

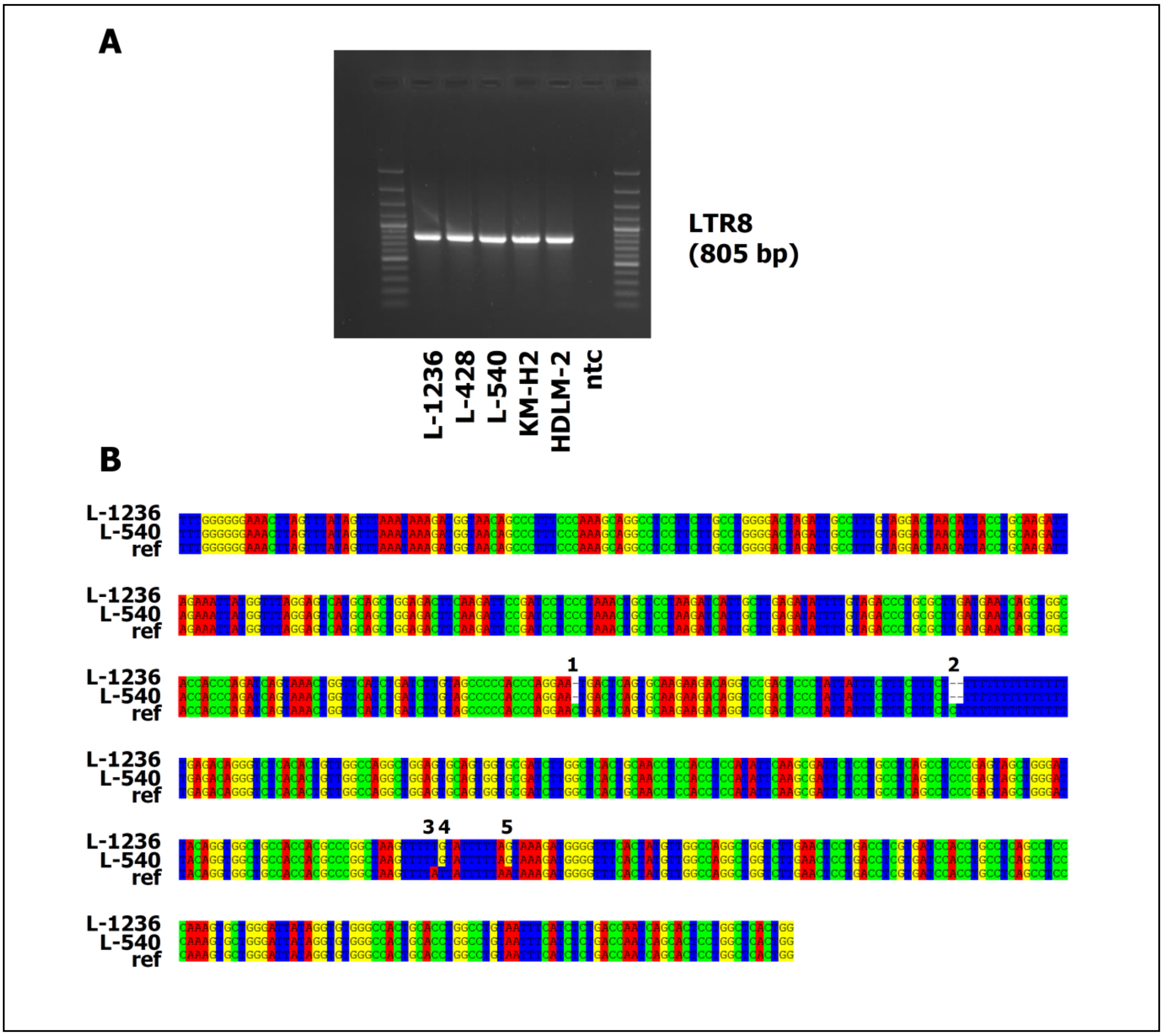

Supplementary Figure 1. Comparison of the THOLE associated LTR8 from Hodgkin lymphoma cell lines and the reference genome sequence. A) Genomic DNA from HL cell lines was used as template for PCR with primers specific for the THOLE-associated LTR8. **B)** PCR products were isolated and sequenced by using the same primers. Presented is an alignment between the sequences from L-1236 and L-540 cells and the genomic reference sequence (NC_000001.11: 109831421-109832103). The indicated differences between the reference sequence and the sequences from HL cell lines are known polymorphisms (1: rs35632747, allele frequency for delC=0.6626; 2: rs71580506, delCT=0.4075; 3: rs569574, T=0.6851; 4: rs557452, G=0.6833; 5: rs569544, G=0.6841; gnomAD data base, European population, 2019). The alignment was visualized with GeneDoc (https://github.com/karlnicholas/GeneDoc, last accessed date: 24. October 2019).

5. Supplementary Figure 2

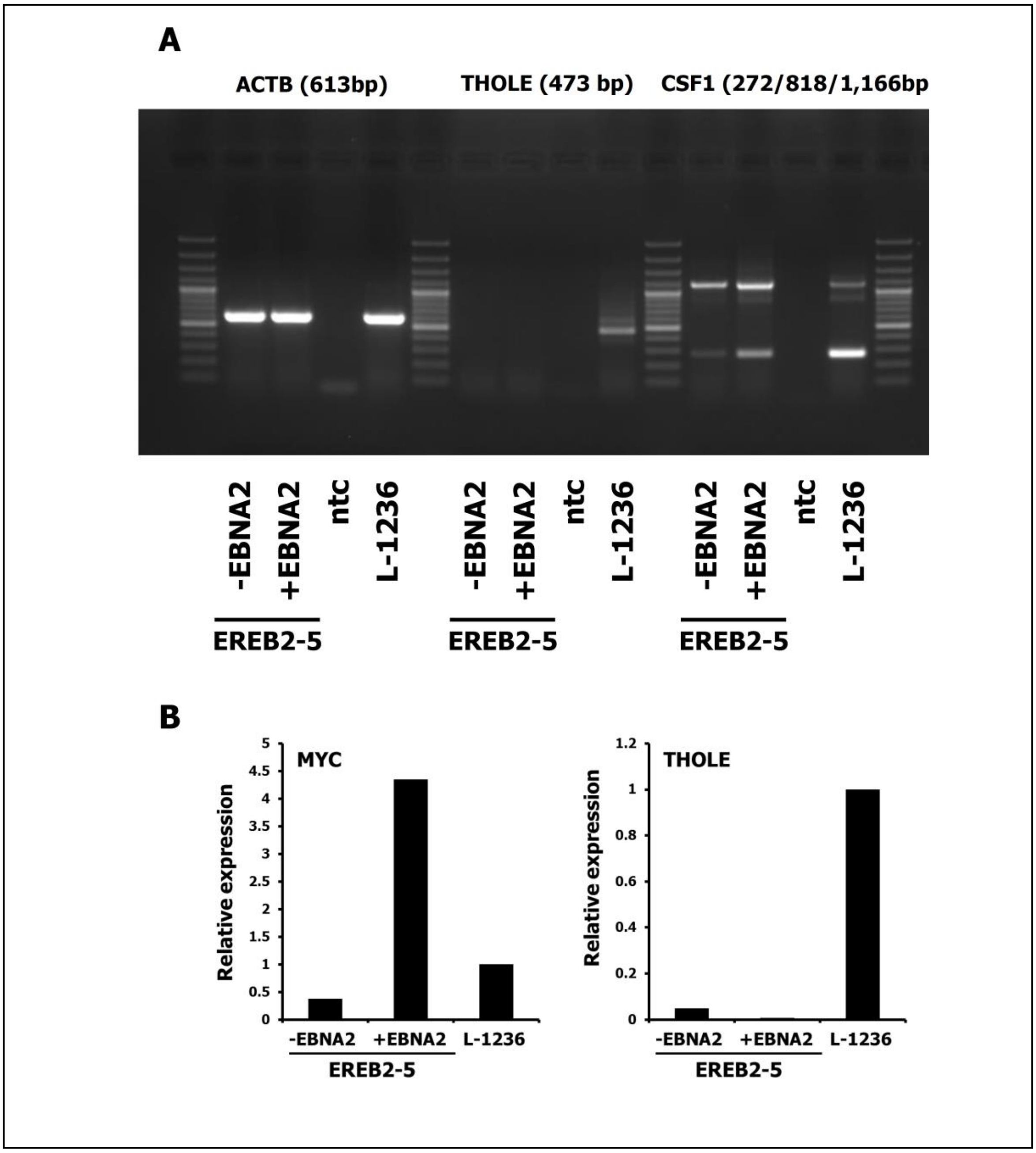

Supplementary Figure 2. Absence of THOLE expression in EBV immortalized cells. Presented are representative results from RT-PCR with the indicated primer combinations. cDNA from L-1236 cells as well as EREB2-5 cells was used as template for RT-PCR. EREB2-5 cells were cultured in the presence of Estrogen (+EBNA2) or in the absence of Estrogen (-EBNA2) for 4 days. Actin beta (ACTB) served as housekeeping control; ntc := no template control. **A)** Conventional PCR. For detection of CSF1 the primer combination "CSF1.V1-4.E5s+E7as" was used. The CSF1 product sizes correspond to transcript variants 3 (272 bp), 1 & 4 (1,166 bp) and 2 (818 bp). **B)** Quantitative RT-PCR. Induction of functional EBNA2 in EREB2-5 cells was proven by detection of increased MYC expression. No induction of THOLE was observed.

6. Supplementary Figure 3

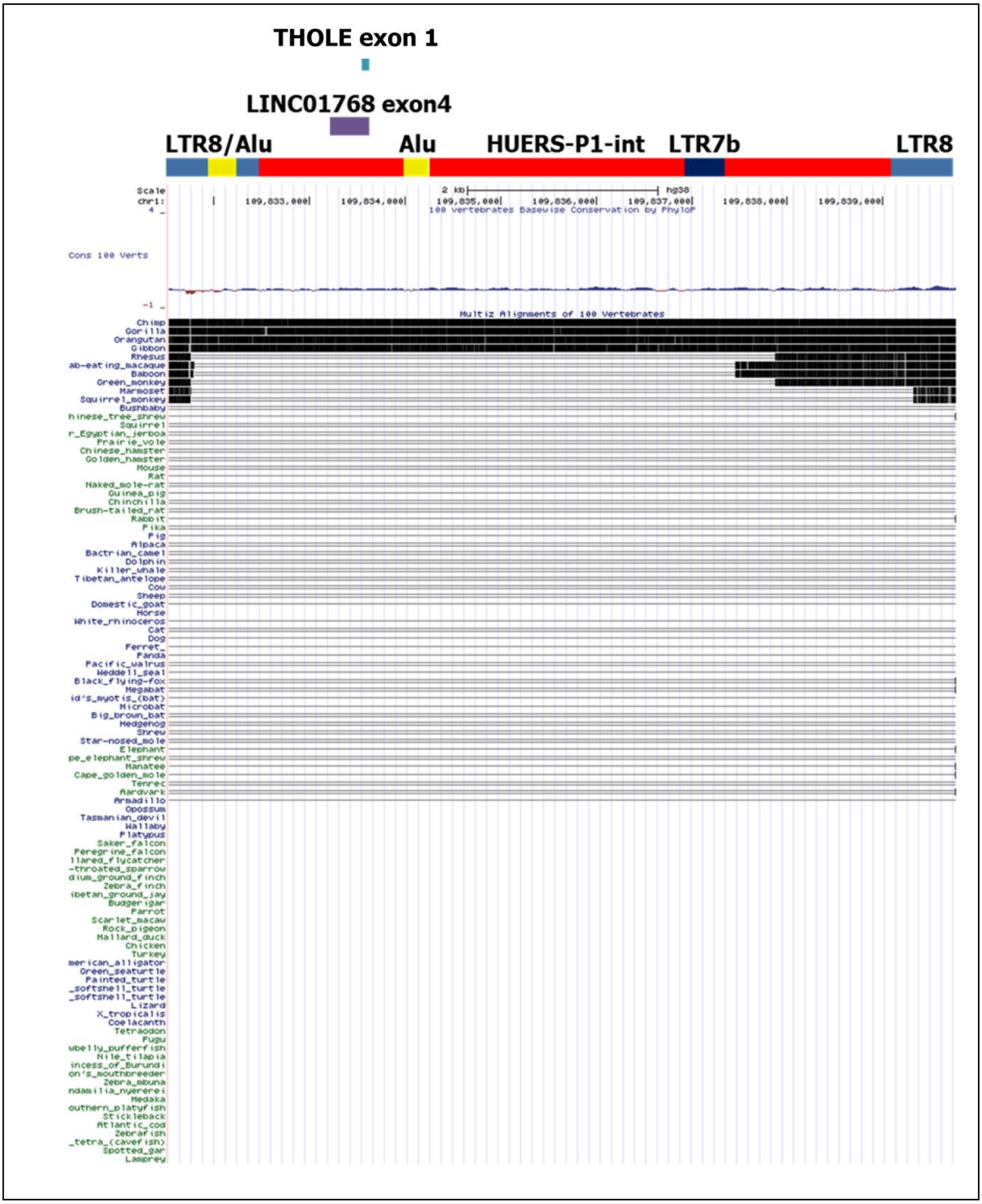

Supplementary Figure 3. Conservation of the THOLE associated ERV in Hominoidea. Conservation of the genomic region surrounding the first THOLE exon (human chr. 1:109,831,532-109,839,764; genome version: hg38) including the LTR8 was visualized with the UCSC genome browser (http://genome.ucsc.edu, last accessed date: 24. October 2019). The positions of exons and repeats are indicated.

6. Supplementary Figure 4

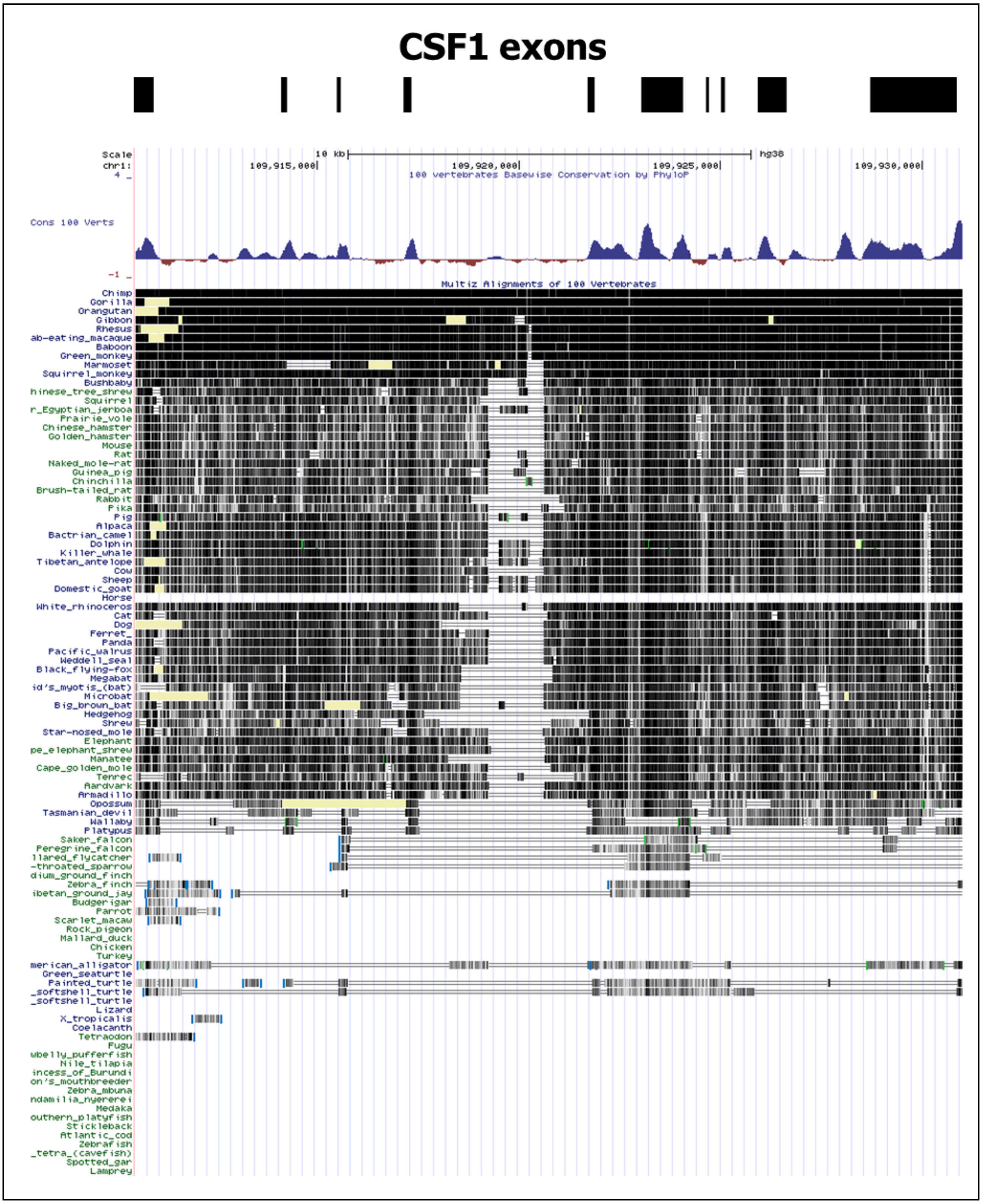

Supplementary Figure 4. Conservation of the CSF1 locus in mammals. Conservation of the CSF1 locus (chr. 1:109,910,506-109,930,992; genome version: hg38) was visualized with the UCSC genome browser (http://genome.ucsc.edu, last accessed date: 24. October 2019)). The positions of exons from CSF1 are indicated.

8. Supplementary Figure 5

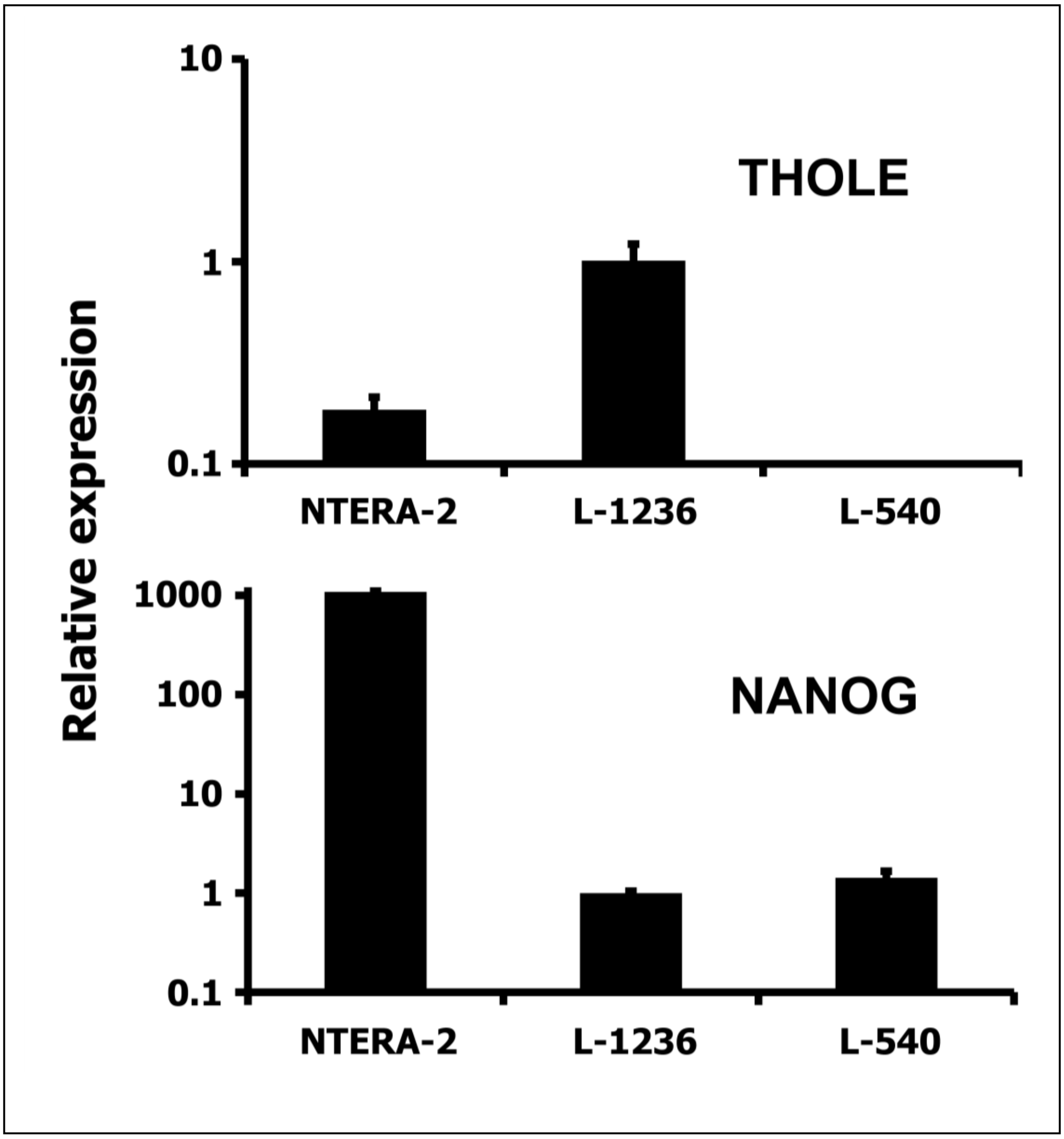

Supplementary Figure 5. Moderate expression of THOLE in embryonic carcinoma cells NTERA-2. Presented are qRT-PCR results with the indicated primer combinations for THOLE and NANOG. cDNA from L-1236 and L-540 HL cells as well as NTERA-2/D1 cells was used as template for qRT-PCR. Actin beta served as housekeeping control. The embryonic gene expression profile of NTERA-2 cells was proven by detection of high NANOG levels. In contrast, only weak THOLE expression was observed.

9. Supplementary Figure 6

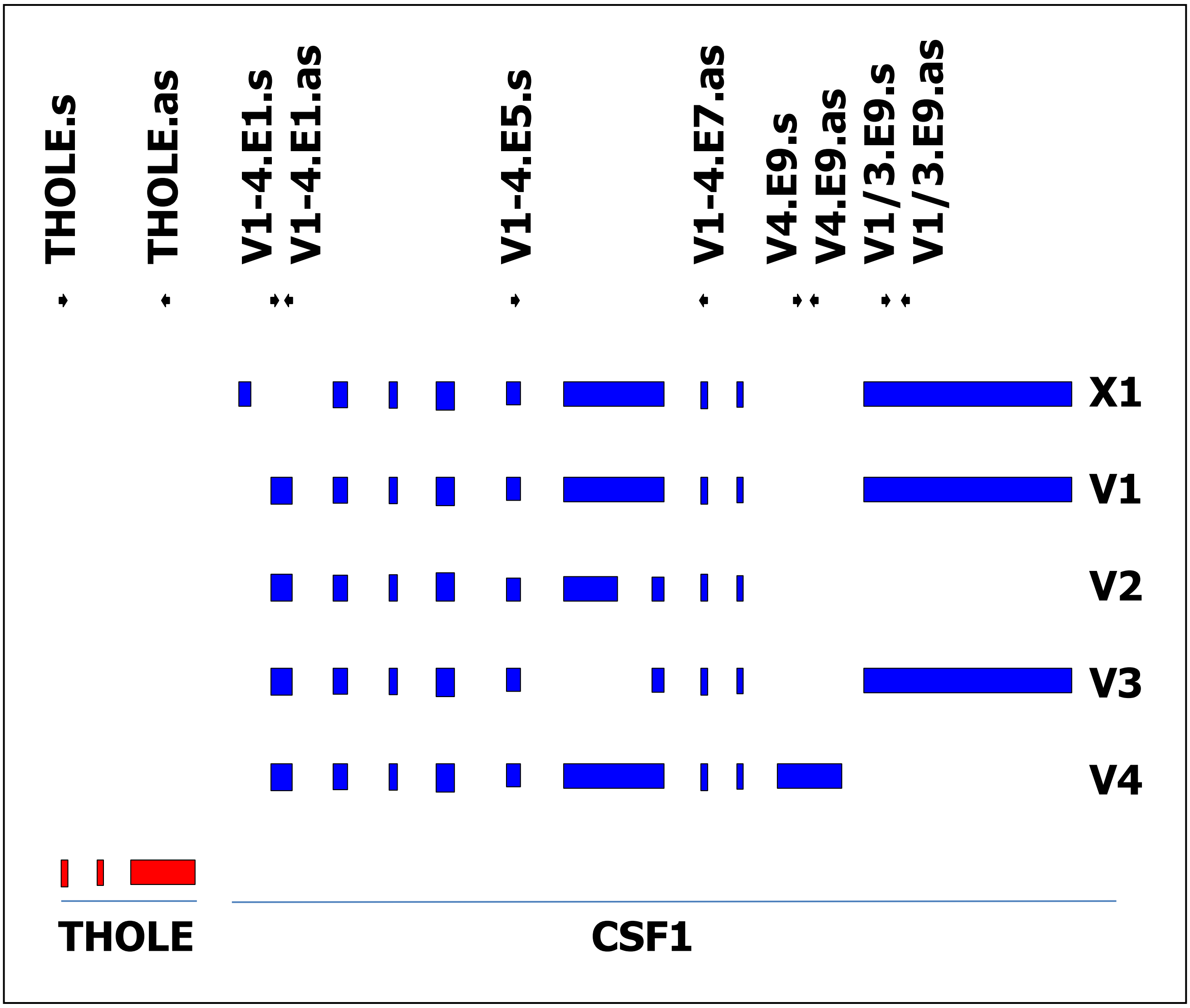

Supplementary Figure 6. Schematic representation of the THOLE/CSF1 region with primer locations. THOLE exons are presented as red boxes, CSF1 exons from transcript variants 1, 2, 3, 4, and X1 are presented as blue boxes. The positions of primers are indicated.
